# The Inflammatory Kinase MAP4K4 Promotes Reactivation of Kaposi's Sarcoma Herpesvirus and Enhances the Invasiveness of Infected Endothelial Cells

**DOI:** 10.1371/journal.ppat.1003737

**Published:** 2013-11-07

**Authors:** Darya A. Haas, Kiran Bala, Guntram Büsche, Magdalena Weidner-Glunde, Susann Santag, Semra Kati, Silvia Gramolelli, Modester Damas, Oliver Dittrich-Breiholz, Michael Kracht, Jessica Rückert, Zoltan Varga, György Keri, Thomas F. Schulz

**Affiliations:** 1 Institute of Virology, Hannover Medical School, Hannover, Germany; 2 Institute of Pathology, Hannover Medical School, Hannover, Germany; 3 Institute of Physiological Chemistry, Hannover Medical School, Hannover, Germany; 4 Rudolf-Buchheim-Institute of Pharmacology, Justus-Liebig-University Giessen, Giessen, Germany; 5 Vichem Chemie Research Ltd., Budapest, Hungary; 6 Department of Medical Chemistry, Semmelweis University, Budapest, Hungary; University of North Carolina at Chapel Hill, United States of America

## Abstract

Kaposi's sarcoma (KS) is a mesenchymal tumour, which is caused by Kaposi's sarcoma herpesvirus (KSHV) and develops under inflammatory conditions. KSHV-infected endothelial spindle cells, the neoplastic cells in KS, show increased invasiveness, attributed to the elevated expression of metalloproteinases (MMPs) and cyclooxygenase-2 (COX-2). The majority of these spindle cells harbour latent KSHV genomes, while a minority undergoes lytic reactivation with subsequent production of new virions and viral or cellular chemo- and cytokines, which may promote tumour invasion and dissemination. In order to better understand KSHV pathogenesis, we investigated cellular mechanisms underlying the lytic reactivation of KSHV. Using a combination of small molecule library screening and siRNA silencing we found a STE20 kinase family member, MAP4K4, to be involved in KSHV reactivation from latency and to contribute to the invasive phenotype of KSHV-infected endothelial cells by regulating COX-2, MMP-7, and MMP-13 expression. This kinase is also highly expressed in KS spindle cells *in vivo*. These findings suggest that MAP4K4, a known mediator of inflammation, is involved in KS aetiology by regulating KSHV lytic reactivation, expression of MMPs and COX-2, and, thereby modulating invasiveness of KSHV-infected endothelial cells.

## Author Summary

Kaposi's sarcoma (KS) is a tumour caused by Kaposi's sarcoma herpesvirus (KSHV) and dysregulated inflammation. Both factors contribute to the high angiogenicity and invasiveness of KS. Various cellular kinases have been reported to regulate the KSHV latent-lytic switch and thereby virus pathogenicity. In this study, we have identified a STE20 kinase family member – MAP4K4 – as a modulator of KSHV lytic cycle and invasive phenotype of KSHV-infected endothelial cells. Moreover, we were able to link MAP4K4 to a known mediator of inflammation and invasiveness, cyclooxygenase-2, which also contributes to KSHV lytic replication. Finally, we could show that MAP4K4 is highly expressed in KS lesions, suggesting an important role for this kinase in tumour development and invasion.

## Introduction

Kaposi's sarcoma (KS) is a mesenchymal tumour caused by Kaposi's sarcoma herpesvirus (KSHV) [1], which originates from blood and lymphatic vessels and develops under the influence of inflammatory cytokines [2]–[4]. Local or systemic inflammation and immunosuppression are important additional risk factors [5], [6]. In addition to KS, KSHV is involved in the pathogenesis of primary effusion lymphoma (PEL) [7], and the plasma cell variant of multicentric Castleman's disease (MCD) [8].

KS is characterised by multiple patch, plaque or nodular lesions on the skin of the extremities or involving the mucosa and visceral organs [9]. KSHV-infected spindle cells, which were shown to be of vascular or lymphatic endothelial origin, represent the main proliferative element in KS and are the distinctive histological signature of advanced KS tumours [10], [11]. The lesions also contain slit-like neovascular spaces, which represent aberrant new vessels [5], [12]. KS spindle cells were shown to have increased invasiveness [13], which has been attributed to the enhanced expression of several matrix metalloproteinases (MMPs) [14], including MMP-1, MMP-2, MMP-3, MMP-7, MMP-9, and MMP-13 [13], [15], [16]. MMPs are zinc-dependent endopeptidases involved in extracellular matrix remodelling during tumour progression, invasion and metastasis [17], [18]. In addition to MMPs, the key enzyme for inducible prostaglandin synthesis – cyclooxygenase 2 (COX-2) [19] – has also been implicated in KS progression and invasion [20]. Increased COX-2 expression in inflammation-driven tumours contributes to neoangiogenesis and activates MMPs, which promote invasiveness [21], [22]. COX-2 is highly expressed in KS tumour tissue and is involved in KS pathogenesis [20], [23], [24]. Several KSHV proteins were shown to enhance COX-2 expression, including K15 [25], and vGPCR [26]. This could explain how KSHV may increase *COX-2* gene expression.

In KS tumours, the majority of KSHV-infected cells harbour latent viral genomes, which are characterised by a restricted viral gene expression pattern that involves the major latent nuclear antigen LANA, homologues of a cellular D-type cyclin and a FLICE inhibitory protein, v-Cyclin and v-FLIP, respectively, and 12 viral miRNAs [6], [27]. However, a minority of infected cells show evidence of productive (‘lytic’) replication and produce not only new virions [28], but also secrete viral or cellular cyto- or chemokines [6], [10], [27], [29], [30]. These are thought to promote the pathological angiogenesis typical for KS lesions, increased invasion, and tumour dissemination [31]. Epidemiological findings also indicate that the prophylactic use of ganciclovir, which inhibits KSHV lytic replication, may reduce the incidence of KS in AIDS patients [32]. In addition, it is thought that the long-term persistence of KSHV *in vivo* may require periodic reactivation from latency and reinfection of new cells [33]. Experimentally, reactivation of KSHV from latency can be initiated by various chemical agents: these include phorbol esters and histone deacetylase inhibitors, which lead to chromatin remodelling and activation of the viral replication and transcription activator (RTA) [34]–[37]. So far, several signalling pathways were reported to be involved in the reactivation of KSHV from latency: PKCδ [38], b-Raf/MEK/ERK [39], PKA [40], Notch and RBP-Jκ [41], [42], p38 and JNK [43], Pim-1 and Pim-3 [44], PI3K and Akt [45], TLR7/8 signalling [46] and others.

Given the importance of the KSHV lytic cycle in KS pathogenesis and the angiogenic and invasive phenotype of KSHV infected cells, we aimed at identifying ‘druggable’ cellular kinases required for KSHV reactivation from latency. To this end, we screened a library of kinase inhibitors and found the STE20 kinase family member MAP4K4 to be a novel mediator of KSHV lytic reactivation. MAP4K4 is known to play an important role in inflammation, insulin resistance, and invasiveness of several malignancies [3], [47]–[55]. We found that MAP4K4 regulates the expression of COX-2, MMP-7 and -13, and thereby modulates the invasiveness of KSHV infected primary and immortalized endothelial cells. Moreover, we found MAP4K4 to be strongly expressed in KSHV-infected endothelial spindle cells in KS tissue, consistent with a role of MAP4K4 in KS pathogenesis.

## Results

### MAP4K4 promotes reactivation of KSHV from latency

**Figure 1:**
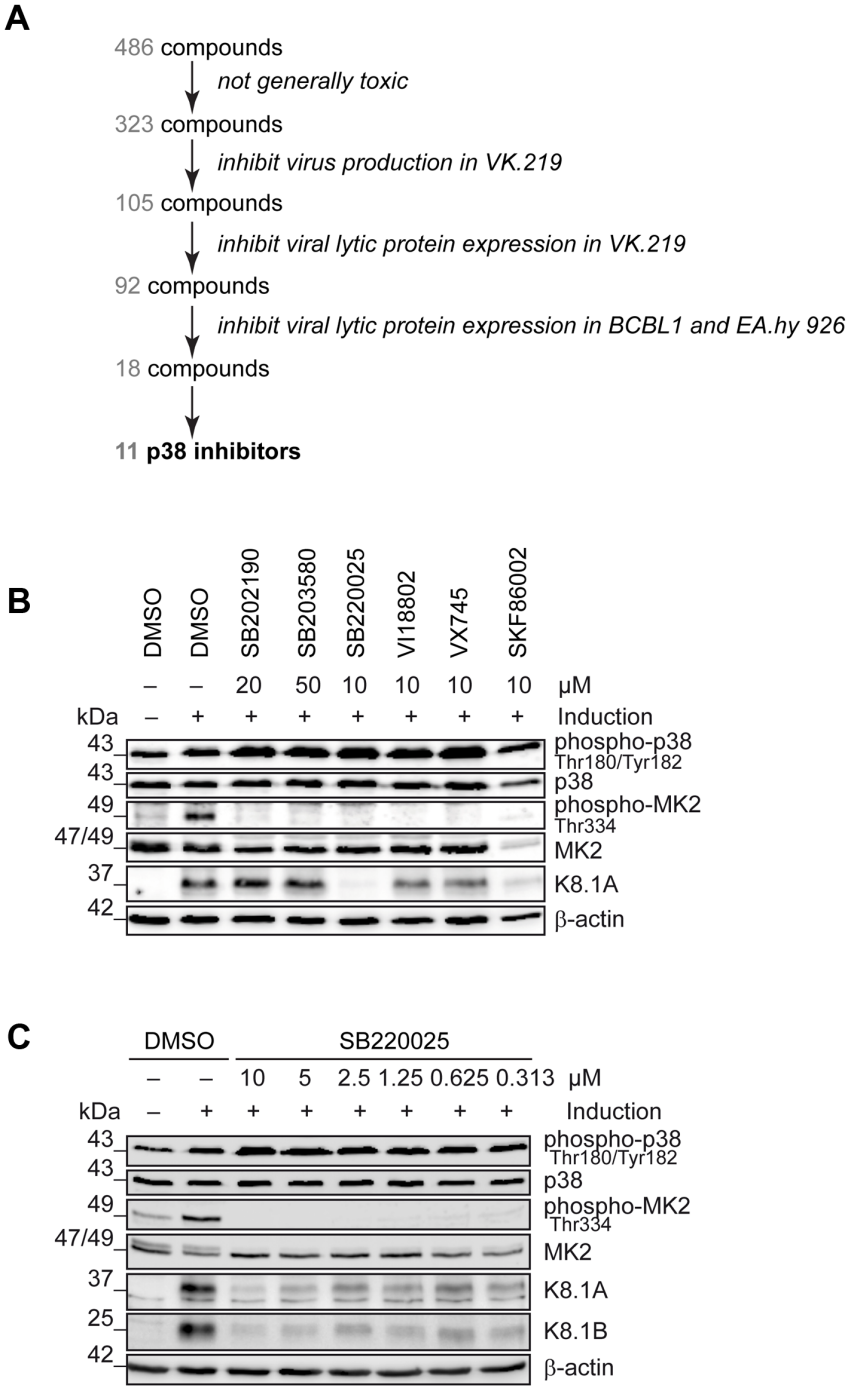
Identification of cellular kinases involved in KSHV reactivation. (A) Schematic of the screen performed to identify cellular kinases important for KSHV lytic reactivation. In total, 486 compounds were applied to VK.219 and HEK293 cells to exclude toxic substances, 323 were screened for inhibition of KSHV production after lytic cycle induction in VK.219 cells, 105 were tested in Western blot analysis for inhibition of KSHV lytic protein expression in VK.219 cells, 92 were validated in Western blot analysis for inhibition of KSHV lytic protein expression in BCBL1 and EA.hy rKSHV.219 cells, out of which 18 compounds proved to be efficiently blocking KSHV production and lytic protein expression, without being generally toxic. 11 of them were known to target p38 MAPK or derived from p38 inhibitors. (B) Effect of p38 inhibitors on KSHV lytic protein expression. EA.hy rKSHV.219 cells were treated with p38 inhibitors at the indicated concentrations one hour prior to the lytic cycle induction. Forty-eight hours after lytic reactivation the cells were lysed and analysed for phospho- and total protein expression. Western blot analysis shows phospho- and total p38 and MK2 expression, and KSHV lytic protein K8.1. The blot is one representative of three independent experiments with similar results. (C) Titration of SB220025 in EA.hy rKSHV.219 cells. EA.hy rKSHV.219 cells were treated with the indicated concentrations of SB220025 or DMSO as a vehicle control one hour before lytic cycle induction. Forty-eight hours after lytic reactivation the cells were analysed for phospho- and total p38, MK2 and K8.1 protein expression. The blot is one representative of three independent experiments with similar results.

Productive replication of KSHV in infected individuals is thought to contribute to viral persistence and the pathogenesis of this virus [56], [57]. Activation of several cellular kinases, involved in different signalling pathways, promotes viral reactivation [58], [59]. In order to identify novel “druggable” cellular kinases required for KSHV reactivation we screened a library of 486 small molecule kinase inhibitors (**figure 1A**) in a KSHV reactivation assay based on Vero cells infected with the recombinant KSHV strain rKSHV.219 (VK.219) [60]. The activation of productive replication cycle was achieved by treatment with Na-butyrate and infection with a baculovirus expressing KSHV immediate-early protein RTA. Toxicity of the compounds was determined by crystal violet staining of VK.219 and HEK293 cells after treatment. As a result, 105 compounds showed moderate to strong effects on virus production and infectivity without being toxic. Among them, 92 compounds were able to directly inhibit KSHV lytic protein expression in VK.219 cells. The results were validated in BCBL1 [61], and KSHV-infected EA.hy 926 [62] cells. As a result, we identified 18 compounds able to inhibit KSHV lytic protein expression in all three cell lines (**figure S1A**). Interestingly, among them were 11 compounds identical to, or derived from, known p38 MAP kinase inhibitors, in line with earlier reports on the role of this kinase in KSHV reactivation [43], [58]. When comparing the effects of commercially available p38 inhibitors with compounds in the VICHEM library, we noted that p38 inhibitors SB202190, SB203580, VX745, SKF86002, SB220025, and a derivative of SB220025 (VI18802) differed in their ability to block KSHV reactivation, as shown by their effect on the expression of KSHV envelope glycoprotein K8.1 (**figure 1B**), although their ability to inhibit the phosphorylation of MK2, a p38 target, seemed comparable (**figure 1B**). Of these compounds, SB220025 was the most potent with regard to inhibiting K8.1 expression (**figure 1B**) or virus production (not shown). To validate the effect of SB220025 on KSHV reactivation, we titrated this compound in KSHV-infected endothelial cells (KSHV-infected EA.hy 926) and found it to inhibit KSHV reactivation at submicromolar concentrations (**figure 1C**). We then determined which other cellular kinases are inhibited by SB220025 and its derivative VI18802. Compound SKF86002, although a strong inhibitor of lytic reactivation, was not included in this comparison, as it reduced the levels of total MK2 (**figure 1B**). We used a commercial screening assay that measured the ability of these compounds to compete with an immobilized ligand for binding to a panel of 442 recombinant kinases in an *in vitro* assay (see www.discoverx.com). The list of cellular kinases inhibited by SB220025 and VI18802 is shown in a table presented in **figure S1B**, which also includes previously published data on compounds SB203580, SB202190 and VX745 [63].

To explore if, apart from p38, any of the other kinases inhibited by SB220025 could account for the strong inhibition of KSHV reactivation observed with this compound, we used small molecule inhibitors or siRNAs against CSNK1D, CSNK1E, CSNK1A1L, MINK, CDC2L1/2, JNK1, MAP4K4, STK36 and TNIK (data not shown). As a result of these experiments we identified the upstream MAP kinase MAP4K4 (data not shown), a member of the STE20 kinase family, which has previously been shown to be involved in inflammation, response to LPS, inflammation-dependent insulin resistance of peripheral tissues, and also invasiveness of several types of cancer cells [47], [48], [53], [64], [65].

**Figure 2:**
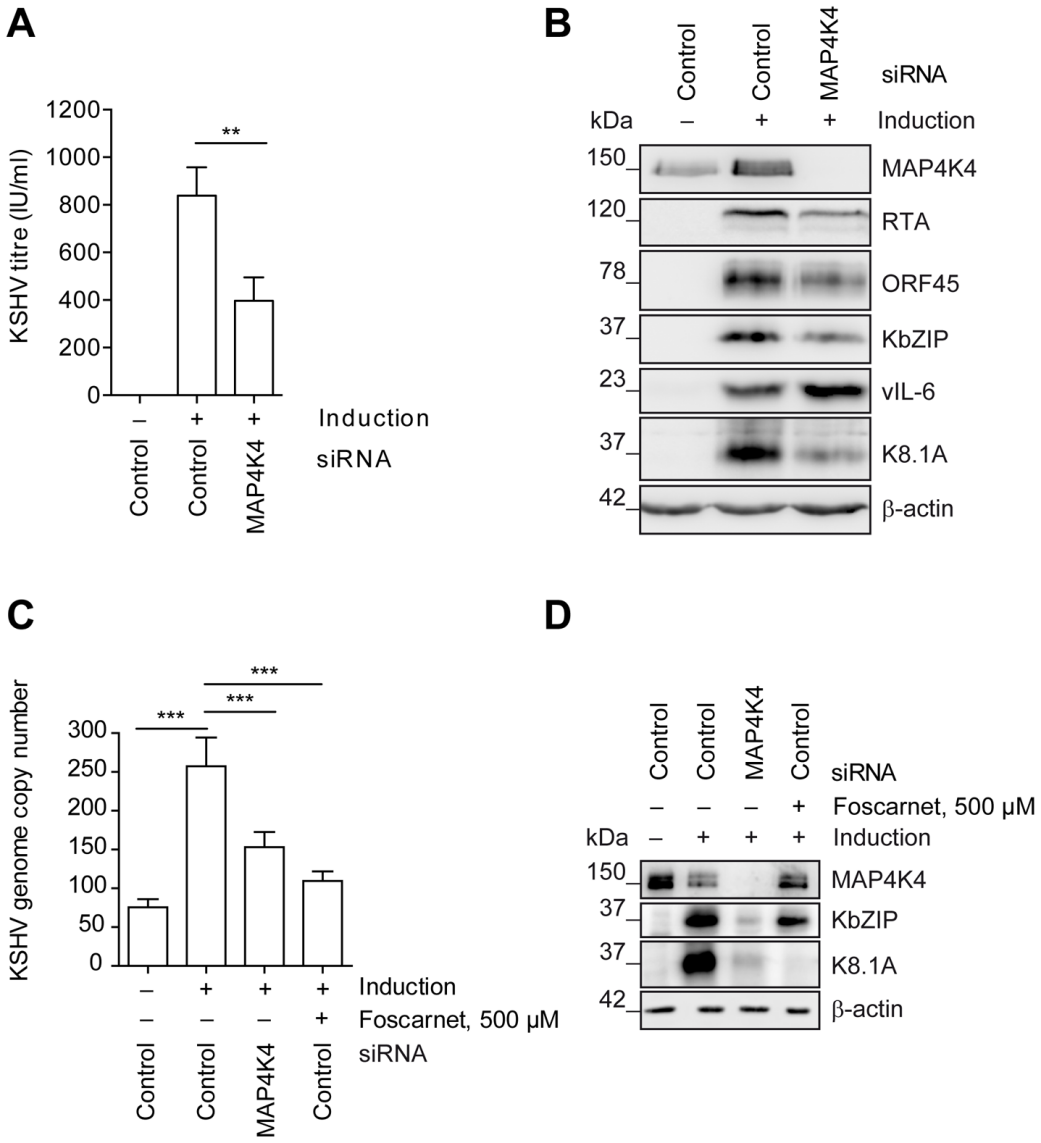
MAP4K4 promotes the lytic reactivation of KSHV. MAP4K4 was silenced with siRNA in EA.hy rKSHV.219 or HuAR2T rKSHV.219 cells twenty-four hours prior to the induction of the viral lytic cycle. Forty-two hours after activating the lytic cycle with Na-butyrate and RTA transduction (see Material and Methods) cells and supernatants were harvested for subsequent analysis. (A) KSHV titre before and after MAP4K4 knockdown. HEK293 cells were infected with viral supernatants from siRNA treated EA.hy rKSHV.219 cells. Production of infectious virus particles was quantified by counting GFP-positive HEK293 cells forty-eight hours after infection. The bar graph shows means ±SD of five independent experiments. The p value was determined using a One-way ANOVA with Tukey's multiple comparison post-test. p<0.01 (**). (B) Western blot analysis of KSHV lytic protein expression. EA.hy rKSHV.219 cells were lysed, cell extracts resolved by SDS-PAGE and the indicated KSHV proteins have been detected with specific antibodies. The blot is one representative of seven independent experiments with similar results. (C) KSHV genome copy number after MAP4K4 depletion or foscarnet treatment. HuAR2T rKSHV.219 cells were lysed, DNA extracted and KSHV genome copy number was evaluated by qPCR analysis. The bar graph shows means ±SD of three independent experiments. The p value was determined using a One-way ANOVA with Tukey's multiple comparison post-test. p<0.001 (***). (D) Western blot analysis of KSHV lytic protein expression after MAP4K4 depletion or Foscarnet treatment. HuAR2T rKSHV.219 cells were lysed, cell extracts resolved by SDS-PAGE and the indicated KSHV proteins detected with specific antibodies. The blot is one representative of three independent experiments with similar results.

In order to explore if MAP4K4 affected KSHV reactivation in a cell type that is known to be infected by KSHV *in vivo*, we used a pool of siRNAs to silence MAP4K4 expression in the immortalized HUVEC derived cell line EA.hy 926 [62], which we had stably infected with rKSHV.219. As shown in **figure 2**, silencing of MAP4K4 in these cells significantly reduced production of infectious viral progeny by more than 60% (**figure 2A**), as well as the expression of immediate-early (RTA), early (KbZIP, ORF45) and late (K8.1) lytic proteins (**figure 2B**). The effect on K8.1 expression was confirmed using four individual siRNAs targeting MAP4K4, all of which were able to reduce MAP4K4 and K8.1 levels (**figure S2A, B**). In contrast to other lytic KSHV proteins, the expression of the viral homologue of IL-6, vIL-6, was slightly increased by MAP4K4 knockdown (**figure 2B**). vIL-6 expression is known to be regulated independently of the productive replication cycle [66] and may therefore not be affected by MAP4K4 silencing. Consistently with the observed decrease in virus production and lytic protein expression, MAP4K4 depletion also reduced KSHV genome replication (**figure 2C**), similarly to foscarnet, an inhibitor of KSHV DNA polymerase [67]. However, while foscarnet only inhibited the expression of a late viral gene (K8.1), MAP4K4 silencing also affected early KSHV gene (KbZIP) expression (**figure 2D**), suggesting that this kinase exerts its effect early in the replication cycle. To control whether MAP4K4 knockdown affects transduction or expression of baculovirus RTA, we evaluated the levels of *RTA* mRNA transcripts before and after MAP4K4 depletion in cells not infected with KSHV that had been treated with baculovirus RTA alone or in combination with Na-butyrate. In these cells, RTA expression was not dependent on MAP4K4 presence (**figure S2C–D**). Taken together, the observed decrease in KSHV titre, lytic protein expression, and replication in the absence of MAP4K4 suggests that this kinase contributes to the successful completion of the KSHV lytic programme.

### MAP4K4 is required for the invasiveness of endothelial cells

**Figure 3:**
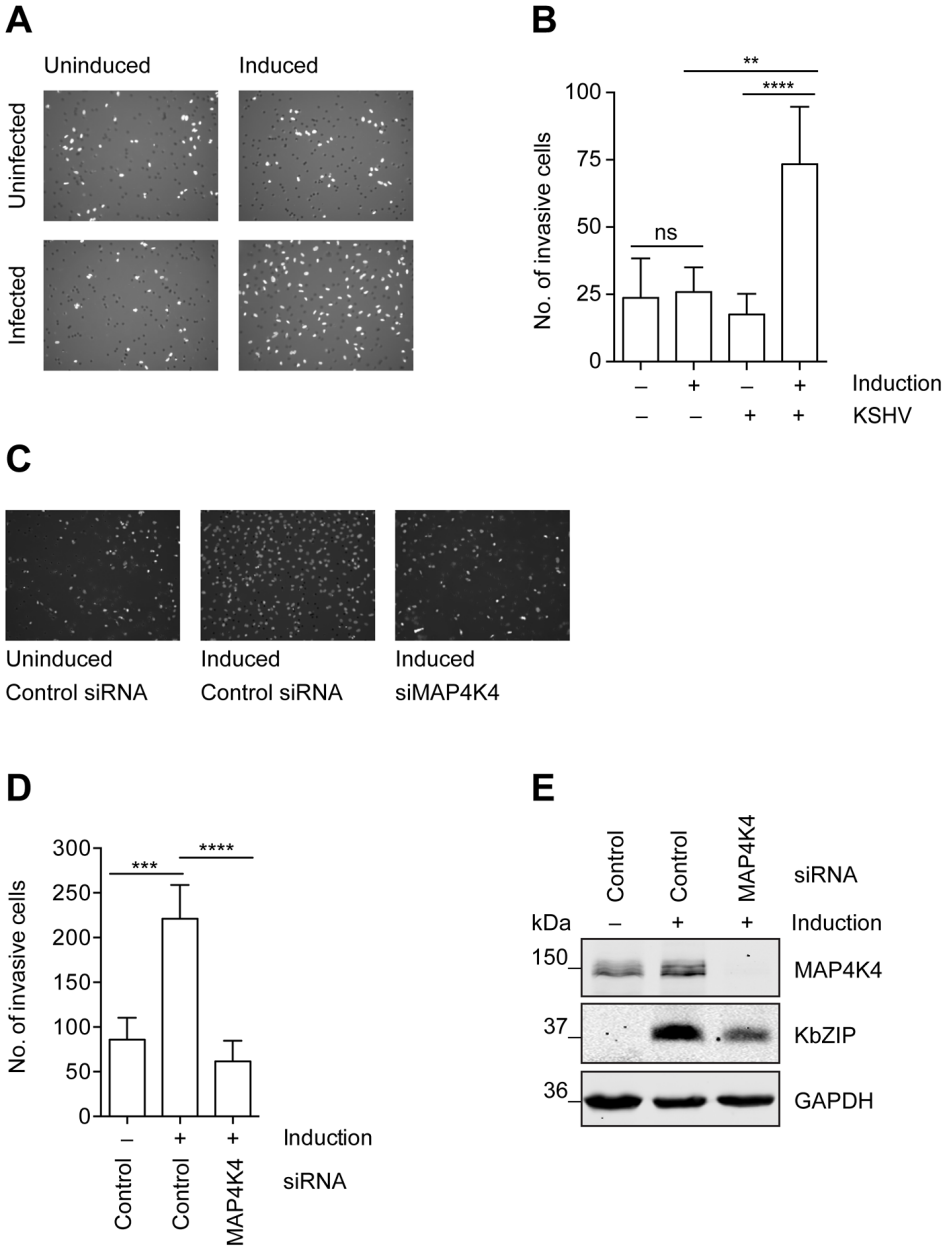
MAP4K4 is required for the increased invasiveness of KSHV-infected endothelial cells. Uninfected HuAR2T cells, or HuAR2T cells stably infected with rKSHV.219 were treated with Na-butyrate and a baculovirus vector expressing RTA, or left untreated, for twenty-four hours with subsequent starving for twelve hours in EBM2 basal medium supplemented with 2% FBS. Equal numbers of cells were seeded on growth factor reduced Matrigel invasion chambers. After twenty-four hours invaded cells were fixed and stained with DAPI. (A) Representative images of invasive cells before and after induction of the lytic cycle. (B) Invasion score measured by quantification of DAPI signal from invasive cells. The bar graph shows means ±SD of invasion scores of three independent experiments. (C) Representative images of invasive KSHV-infected cells before and after MAP4K4 depletion and after induction of the lytic replication cycle. MAP4K4 or control siRNA were transfected twenty-four hours prior to the induction of the lytic replication cycle. (D) Invasion score presented as means ±SD of three independent experiments. The p values were determined using a One-way ANOVA with Tukey's multiple comparison post-test. p>0.05 (ns); p<0.05 (*); p<0.01 (**); p<0.001 (***); p<0.0001 (****). (E) Western blot analysis of MAP4K4 and KSHV KbZIP early lytic protein expression.

MAP4K4 is also known to promote tumour cell migration, invasion, and loss of adhesion [49], [68]. KS tumour derived cells have been reported to show an invasive phenotype [69]. This phenomenon can be studied *in vitro* in a matrigel-based invasion assay, in which uninfected HuAR2T, a conditionally immortalized HUVEC cell line [70], fails to invade into matrigel, whereas HuAR2T cells infected with rKSHV.219 show increased invasiveness after the treatment with Na-butyrate to induce the KSHV lytic replication cycle (**figure 3A–B**). Thus, lytic reactivation of the virus promotes invasiveness of these immortalized endothelial cells infected with KSHV. As we observed that MAP4K4 supports the KSHV lytic cycle (**figure 2**) and since it had been reported to be a promigratory kinase [49], we investigated if its silencing might affect the ability of KSHV-infected endothelial cells to invade matrigel. Indeed, after silencing of MAP4K4 expression with siRNA, KSHV-infected HuAR2T endothelial cells failed to invade matrigel beyond the levels seen in uninfected control cells (**figure 3C–D**). MAP4K4 and KSHV lytic protein expression was controlled by Western blot analysis as presented in **figure 3E**. Together, these data suggest a role for MAP4K4 signalling in the KSHV-dependent invasiveness of infected endothelial cells.

### Identification of genes whose expression is regulated by MAP4K4 signalling

**Figure 4:**
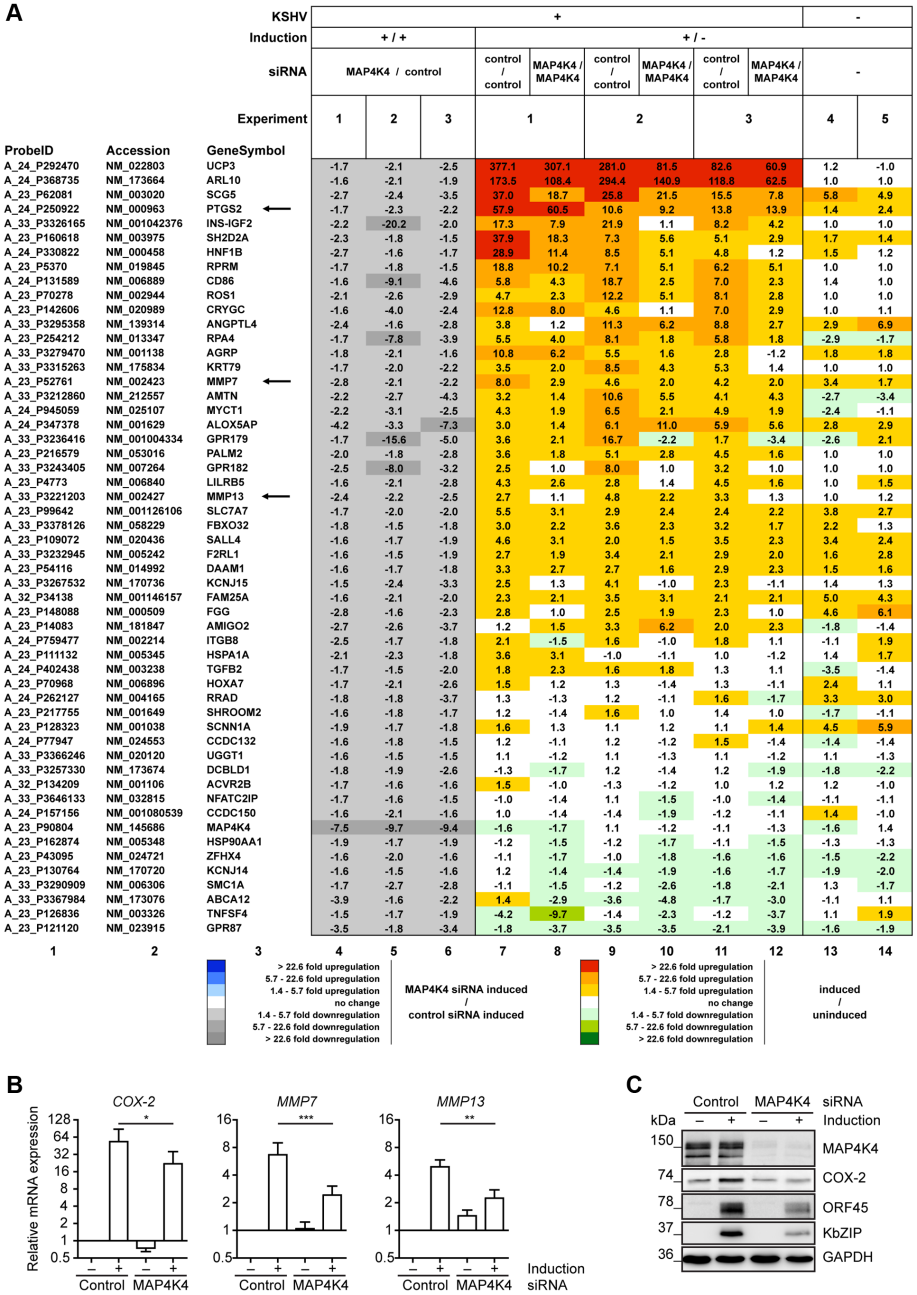
Identification of cellular genes regulated by MAP4K4 signalling in KSHV-infected endothelial cells. HuAR2T rKSHV.219 cells were transfected with control siRNA or siRNA targeting MAP4K4 twenty-four hours before the induction of the lytic cycle. Cells were harvested twenty-four hours after inducing the lytic cycle. (A) Alterations in cellular gene expression following MAP4K4 knockdown and KSHV lytic reactivation. 54 cellular genes regulated by MAP4K4 (columns 4–6; grey scale) by a factor of >1.5 were identified by comparing their expression in lytically induced HuAR2T rKSHV.219 cells silenced for MAP4K4 expression with control siRNA treated induced cells. The effect of lytic KSHV reactivation on cellular gene expression is shown on a red-green scale in cells treated with either control siRNA (‘control/control’) or MAP4K4 siRNA (‘MAP4K4/MAP4K4’) (columns 7–12). Uninfected HuAR2T cells were analysed in two additional experiments to evaluate the effects of the induction compounds in the absence of KSHV (columns 13–14). Data were ordered according to the average fold induction strength in control siRNA treated HuAR2T rKSHV.219 cells after lytic induction (columns 7, 9, 11). The fold ratios for *PTGS2* presented in columns 7 to 12, which show induced/uninduced ratios from control siRNA treated samples (columns 7, 9, 11) *versus* siMAP4K4 treated samples (columns 8, 10, 12), appear to be similar. This is due to the fact that COX-2 expression is reduced by a similar factor in uninduced and induced samples following MAP4K4 silencing. (B) qPCR analysis of COX-2, MMP-7 and MMP-13 expression in HuAR2T rKSHV.219 using dually labelled probes presented as means ±SD of three independent experiments. The p values were determined using a One-way ANOVA with Tukey's multiple comparison post-test. p>0.05 (ns); p<0.05 (*); p<0.01 (**); p<0.001 (***). (C) Western blot analysis of COX-2 expression before and after MAP4K4 depletion in latent and lytically induced HuAR2T rKSHV.219 cells. The blot is one representative of five independent experiments with similar results.

In an attempt to understand how MAP4K4 promotes lytic reactivation and leads to the increased invasiveness of KSHV-infected endothelial cells we compared the transcriptome of reactivated KSHV-infected HuAR2T cells, in which the expression of MAP4K4 had been silenced with siRNA, with KSHV-infected, reactivated HuAR2T cells treated with control siRNA. We were able to identify 54 cellular genes that showed at least a 1.5-fold decrease in their expression levels after MAP4K4 knockdown in HuAR2T rKSHV.219 undergoing viral reactivation as compared to control siRNA treated, reactivated HuAR2T rKSHV.219 cells in at least two out of three independent experiments (**figure 4A**). Successful knockdown of MAP4K4, and the subsequent inhibition of lytic gene expression, was controlled by Western blot analysis (**figure S2E**). Among the cellular genes regulated by MAP4K4 silencing in KSHV-infected endothelial cells were three that have previously been reported to contribute to the invasive phenotype of tumour cells: *PTGS2*, encoding cyclooxygenase 2 (COX-2), and the genes coding for matrix metalloproteinases 7 and 13 (MMP-7 and MMP-13) (**figure 4A**). In order to validate the results of the transcriptome analysis, the expression levels of COX-2 were evaluated by qPCR and Western blot analysis before and after the induction of the lytic cycle. As shown in **figure 4B–C**, COX-2 mRNA and protein expression is upregulated following induction of the viral lytic cycle and can be reduced by silencing MAP4K4. Likewise, we could show that the expression of both MMP-7 and MMP-13 mRNAs increased after the induction of the lytic cycle and was significantly reduced after MAP4K4 depletion (**figure 4B**). These data support the notion that MAP4K4 may mediate the increased invasiveness of KSHV-infected endothelial cells due to its ability to modulate not only COX-2, but also MMP-7 and MMP-13 expression.

### MMP-7 and MMP-13 are required for KSHV-driven endothelial cell invasiveness

**Figure 5:**
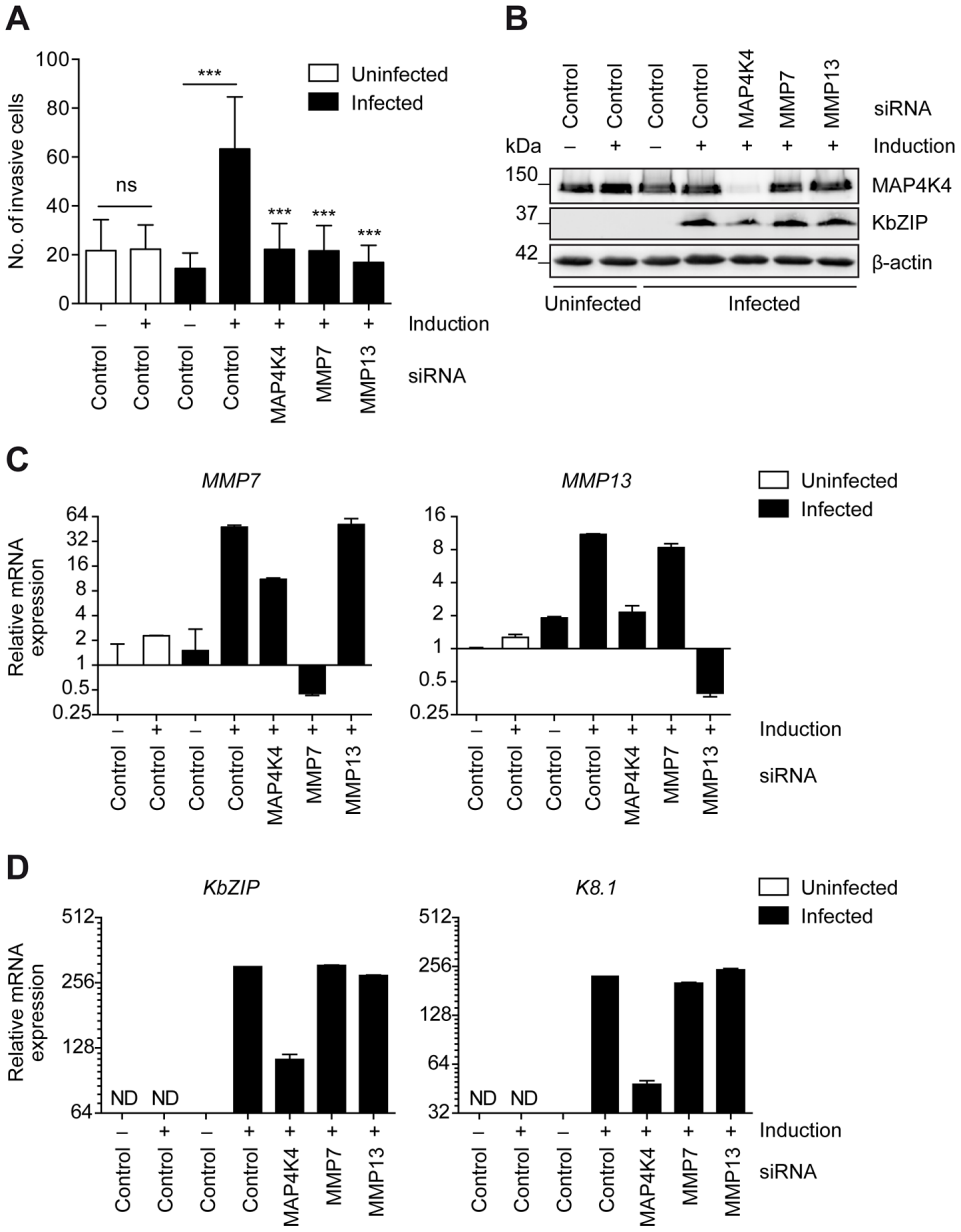
MMP7 and MMP13 are required for the invasiveness of KSHV-infected endothelial cells. HuAR2T or HuAR2T rKSHV.219 cells were transfected with control siRNA or siRNA pools targeting MAP4K4, MMP7 or MMP13 twenty-four hours before the induction of the lytic cycle. Thirty-six hours after lytic reactivation starved cells were analysed for invasiveness. (A) Invasion score determined by quantifying DAPI stained invasive cells during latency and in the course of lytic reactivation and presented as means ±SD of four independent experiments. The p values were determined using a One-way ANOVA with Tukey's multiple comparison post-test. p>0.05 (ns); p<0.05 (*); p<0.01 (**); p<0.001 (***); p<0.0001 (****). (B) Western blot analysis of MAP4K4 and KbZIP expression in HuAR2T and HuAR2T rKSHV.219 cells. The blot is one representative of four independent experiments with similar results. (C) qPCR analysis of *MMP-7* and *MMP-13* expression in HuAR2T and HuAR2T rKSHV.219 cells. The graph is one representative of four independent experiments with similar results. (D) qPCR analysis of *KbZIP* and *K8.1* mRNA expression in HuAR2T and HuAR2T rKSHV.219 cells. The graph is one representative of four independent experiments with similar results.

KS cells are known to express high levels of MMP-1, -2, -3, -7, -9, -13, -19, and previous reports suggest that some of these metalloproteinases may contribute to the invasive phenotype of the tumour [14], [16], [71], [72]. Overexpression of MMP-7 has been reported in several other malignancies [73]–[75], and its depletion with siRNA resulted in a significant decrease in the invasive potential of different cancer cell types [76]–[78]. Similarly, MMP-13 has been reported to confer the ability to penetrate basement membranes and ECM upon malignant cells [79]. Given these proinvasive properties of MMP-7 and MMP-13, and taking into account the ability of MAP4K4 to regulate their expression (**figure 4**), we addressed the involvement of these metalloproteinases in the invasiveness of KSHV-infected cells in a matrigel-based invasion assay. We found that depletion of both MMP-7 and MMP-13, similarly to MAP4K4 knockdown, led to a significant reduction of the number of invasive KSHV-infected endothelial HuAR2T cells following activation of the viral lytic replication cycle (**figure 5A**). The efficiency of silencing the expression of MAP4K4, MMP-7 and MMP-13 with siRNA was controlled by Western blot analysis for MAP4K4 (**figure 5B**) and qPCR for MMP-7 and MMP-13 (**figure 5C**). We noted that silencing of MAP4K4 led to a reduced expression of the early KSHV protein KbZIP (**figure 5B**) and its mRNA transcript, as well as K8.1 mRNA expression (**figure 5D**), whereas silencing of MMP-7 and MMP-13 had no effect on the protein and mRNA levels of KbZIP (**figure 5B, 5D**) or mRNA levels of K8.1 (**figure 5D**). These results suggest that MAP4K4 is involved in the activation of the lytic replication cycle, which, in turn, promotes the expression of MMP-7 and MMP-13.

### MAP4K4-dependent COX-2 expression and enzymatic activity is required for successful reactivation of KSHV and the invasiveness of KSHV-infected endothelial cells

**Figure 6:**
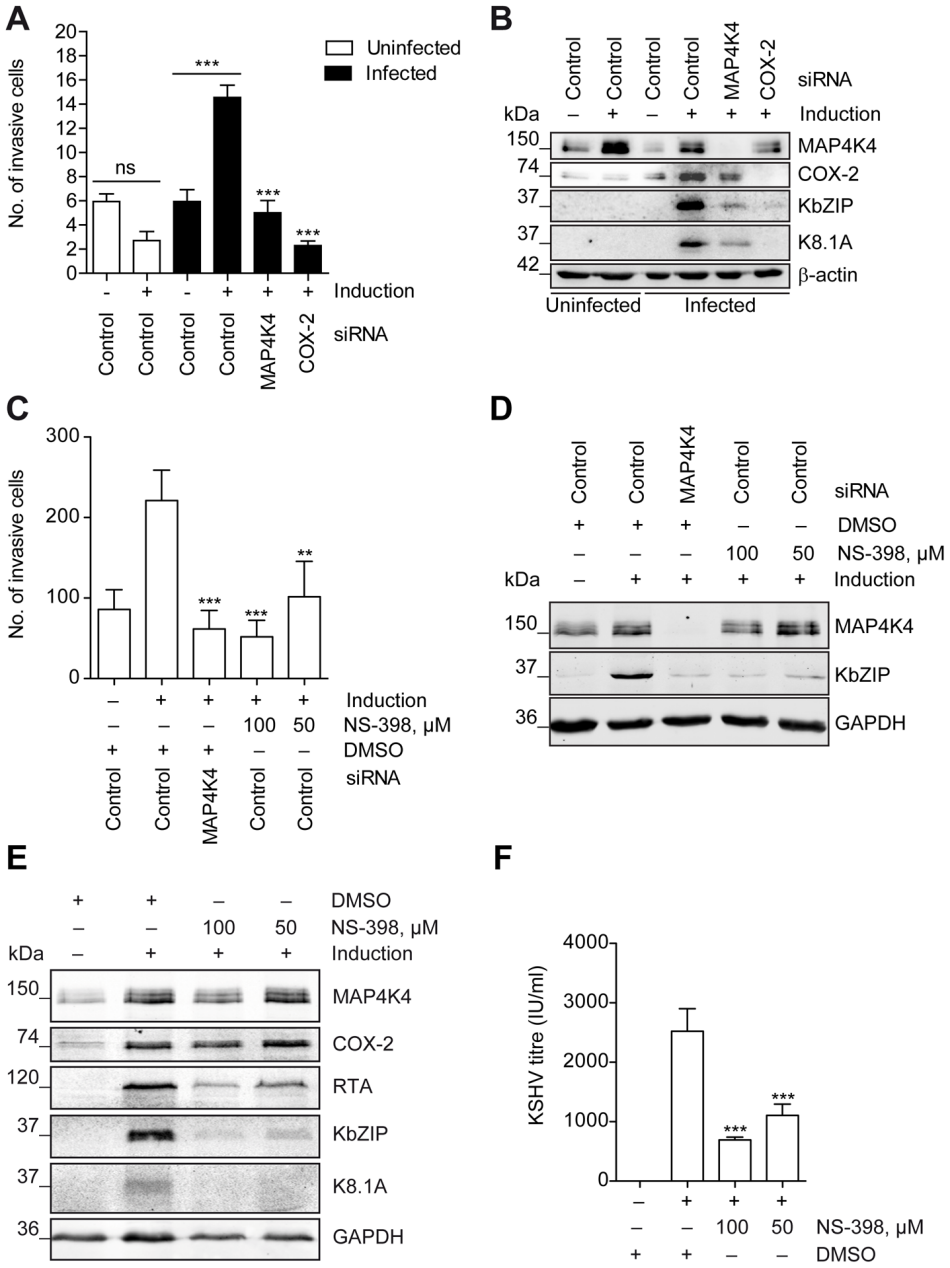
COX-2 enzymatic activity contributes to the successful reactivation of KSHV and the invasiveness of KSHV-infected endothelial cells. HuAR2T rKSHV.219 cells were transfected with control siRNA, MAP4K4- or COX-2 targeting siRNA pools or treated with NS-398 or vehicle control, and subsequently analysed for invasiveness and KSHV lytic protein expression. (A) Invasion score determined after MAP4K4 or COX-2 depletion by quantifying DAPI stained invasive cells. The graph is one representative of two independent experiments with similar results. (B) Western blot analysis of MAP4K4, COX-2, KbZIP, and K8.1 expression. The blot is one representative of two independent experiments with similar results. (C) Invasion score determined after MAP4K4 depletion or COX-2 chemical inhibition by quantifying DAPI stained invasive cells. The graph is one representative of three independent experiments with similar results. (D) Western blot analysis of KSHV lytic protein expression after MAP4K4 silencing or treatment with COX-2 inhibitor NS-398. The blot is one representative of three independent experiments with similar results. (E) Western blot analysis of KSHV lytic protein expression after treatment with COX-2 inhibitor NS-398. The blot is one representative of three independent experiments with similar results. (F) KSHV titre determined by quantifying GFP positive HEK293 cells forty-eight hours after infection with supernatants from untreated or NS-398 treated HuAR2T rKSHV.219 cells. The graph shows means ±SD of three independent experiments. The p values were determined using a One-way ANOVA with Tukey's multiple comparison post-test. p>0.05 (ns); p<0.05 (*); p<0.01 (**); p<0.001 (***); p<0.0001 (****).

As shown in **figure 4**, silencing of MAP4K4 reduces the expression of *PTGS2*, encoding cyclooxygenase 2 (COX-2). COX-2 has previously been shown to be overexpressed in KSHV-infected endothelial cells and to play a role in inflammation, angiogenesis and invasion [20]. The KSHV K15 and vGPCR proteins induce the expression of COX-2 [25], [26]. COX-2 catalyses the production of prostaglandin E_2_ (PGE_2_) after stimulation with inflammatory cytokines [80]. Depletion of COX-2 reduced invasiveness of KSHV-infected endothelial cells, similar to MAP4K4 knockdown (**figure 6A**). Interestingly, both MAP4K4 and COX-2 silencing inhibited KSHV lytic reactivation (**figure 6B**). To corroborate the effect of COX-2 depletion on KSHV lytic reactivation, we used a specific inhibitor, which does not affect constitutively active COX-1 [81]. Application of this inhibitor, NS-398, led to a dramatic decrease, comparable to the effect of MAP4K4 silencing, in the invasiveness of KSHV-infected endothelial cells undergoing lytic reactivation (**figure 6C**). NS-398 treatment not only led to a reduction of invasiveness, but also effectively blocked KSHV lytic protein expression (**figure 6D–E**), as well as the production of viral progeny (**figure 6F**). This suggests that, in response to MAP4K4 signalling, COX-2 mediated production of PGE_2_ contributes to the successful completion of KSHV lytic cycle and KSHV driven invasiveness of infected endothelial cells.

### MAP4K4 contributes to the invasiveness of primary endothelial cells infected with KSHV

**Figure 7:**
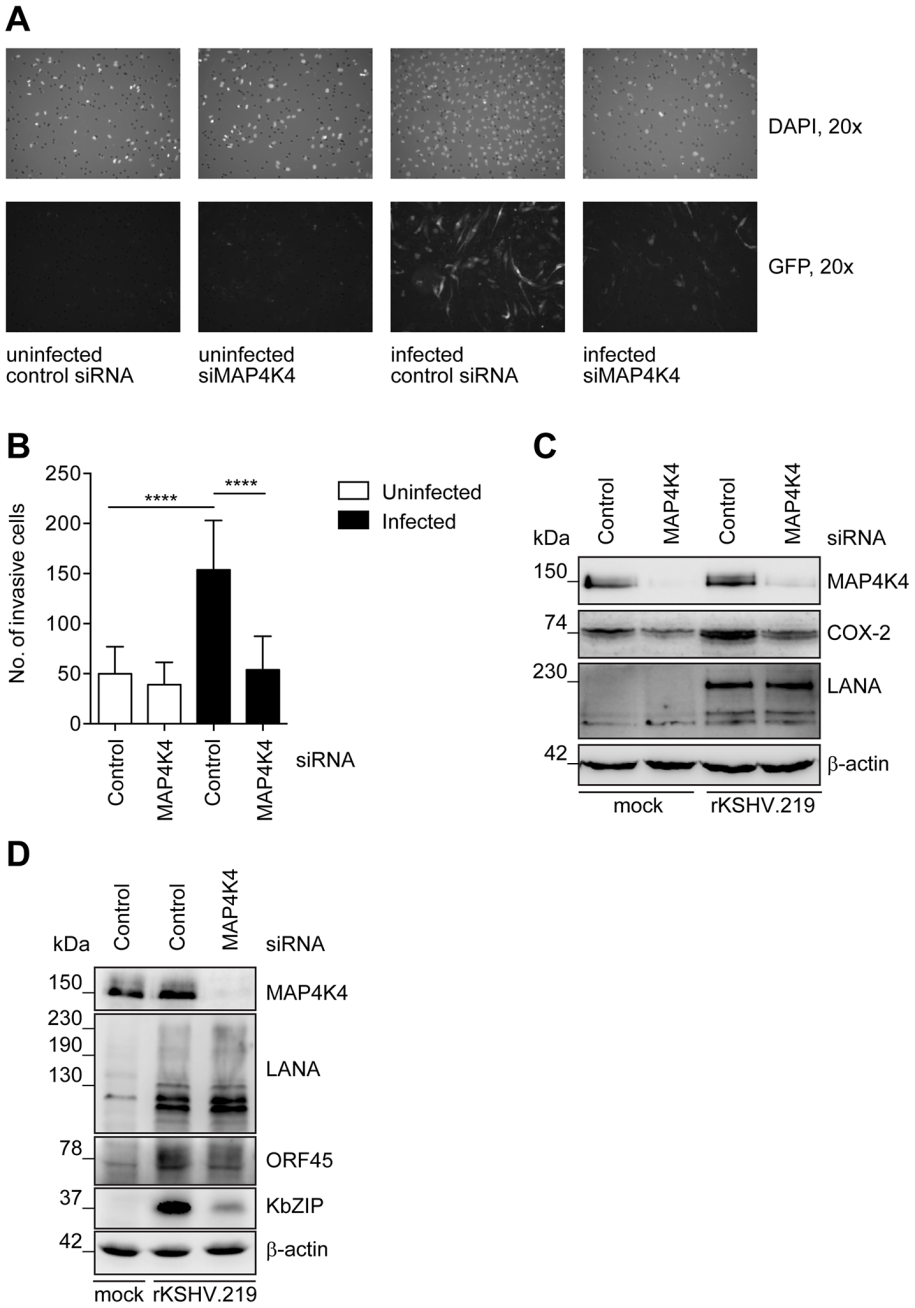
Increased MAP4K4 expression in KSHV-infected primary endothelial cells promotes their invasiveness. HUVEC were infected with concentrated rKSHV.219 at an MOI of 20 and monitored for MAP4K4 and KSHV lytic protein expression and invasiveness. (A) Representative images of invasive uninfected or rKSHV.219-infected HUVEC before and after MAP4K4 depletion. (B) Invasion score determined by quantifying DAPI stained uninfected or rKSHV.219-infected HUVEC, before and after MAP4K4 depletion. The graph shows means ±SD of three independent experiments. The p values were determined using a One-way ANOVA with Tukey's multiple comparison post-test. p>0.05 (ns); p<0.05 (*); p<0.01 (**); p<0.001 (***); p<0.0001 (****). (C) Western blot analysis of MAP4K4, COX-2 and LANA expression in uninfected or infected HUVEC. The blot is one representative of three independent experiments with similar results. (D) Western blot analysis of MAP4K4, LANA, ORF45 and KbZIP protein expression after infection of HUVEC with rKSHV.219 and MAP4K4 depletion.

To extend our observations, which were obtained with the immortalized endothelial cell line HuAR2T, to primary endothelial cells, we investigated the role of MAP4K4 in the invasiveness of human umbilical vein endothelial cells (HUVECs) following their infection with rKSHV.219 (**figure 7**). On day 5 after infection, KSHV-infected HUVECs showed a markedly increased invasiveness compared to uninfected cells, and this increased invasiveness depended on the expression of MAP4K4, since silencing of MAP4K4 with siRNA reduced their invasiveness to background levels (**figure 7A–B**). Similar to KSHV-infected HuAR2T cells, expression of COX-2 increased after infection of HUVECs with KSHV and silencing of MAP4K4 by siRNA reduced COX-2 levels in KSHV-infected primary endothelial cells (**figure 7C**). We also observed that after infection with KSHV, MAP4K4 protein levels were moderately increased (**figure 7C–D**). Moreover, KSHV lytic protein expression was inhibited after MAP4K4 depletion in primary cells, similarly to what we had found in immortalized endothelial cells (**figure 7D**).

### MAP4K4 is expressed in KS spindle cells *in vivo*

**Figure 8:**
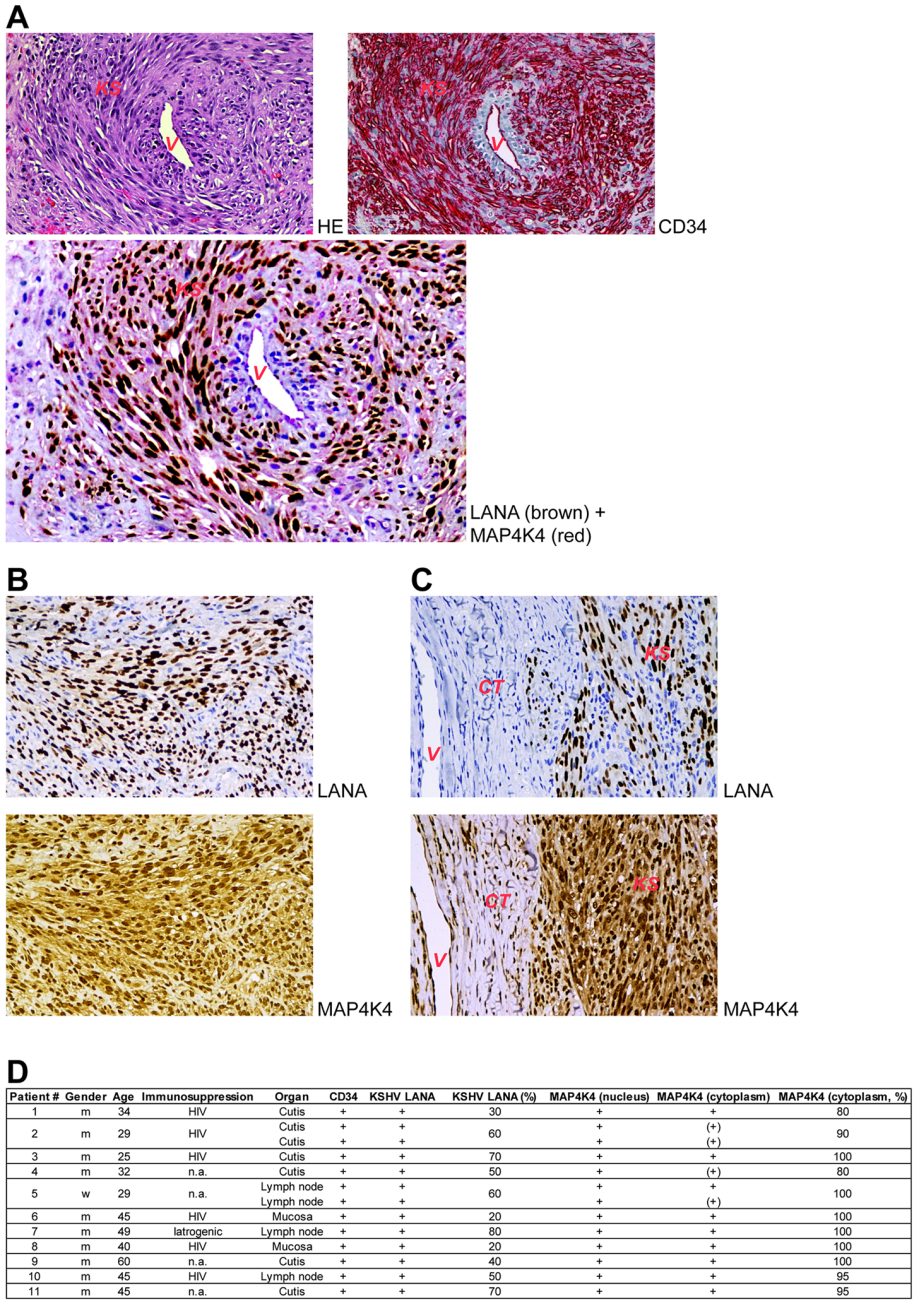
MAP4K4 is expressed in endothelial spindle cells in KS tumours. (A) Subepidermal Kaposi's sarcoma [KS] surrounding and infiltrating a non-neoplastic blood vessel [V]. The tumour cells express CD34 (red; the non-neoplastic endothelial cells of the blood vessel are CD34 positive, too) and nuclear LANA (dark brown), as well as cytoplasmic MAP4K4 (light red; double-staining). (B) Kaposi's sarcoma completely replacing the lymphatic tissue of a lymph node. Nuclear LANA protein expression is observed in 50–70% of tumour cells (dark-brown) and cytoplasmic MAP4K4 expression – in more than 95% of sarcoma cells (brown). (C) Kaposi's sarcoma [KS] partly expressing LANA and infiltrating non-neoplastic subepidermal connective tissue [CT] with a non-neoplastic blood vessel [V]. Strong cytoplasmic MAP4K4 positivity is seen in KS cells, while only weak MAP4K4 expression is present in non-neoplastic endothelial cells [V]. All images are presented in 250× magnification. (D) Summary of MAP4K4 expression in 13 KS biopsies.

To explore if MAP4K4 is expressed in KS tissue and could, therefore, play a role in KSHV-infected cells *in vivo* and contribute to the pathogenesis of KS, we stained KS biopsies with an antibody to MAP4K4. We observed a strong expression of MAP4K4 in the KS endothelial spindle cells, which are characterised by the expression of CD34 and KSHV LANA (**figure 8A**). Double staining for LANA and MAP4K4 confirmed the strong cytoplasmic expression of MAP4K4 in LANA-expressing cells (**figure 8A**). Individual staining for MAP4K4 and LANA of adjacent serial sections of a KS biopsy also indicated the increased expression of MAP4K4 in LANA-expressing KS spindle cells, although a lower level of MAP4K4 expression could also be seen in other cells in the tumour (**figure 8B–C**), and a basal expression of MAP4K4 was observed in the surrounding connective tissue (**figure 8C**), in line with another report showing low levels of MAP4K4 cytoplasmic staining in non-neoplastic lung tissues, compared to strong expression in lung adenocarcinomas [82]. We found a moderate to strong expression of MAP4K4 in spindle cells in a total of 13 biopsies, derived from 11 patients (**figure 8D**), confirming the consistent expression of this kinase in KS tissue. This observation is consistent with a role for MAP4K4 and MAP4K4-dependent signalling pathways in the pathogenesis of KS.

## Discussion

In KS tumours, a small percentage (1–5%) of KSHV-infected cells show evidence of viral lytic replication [31], [83]. Taken together with epidemiological findings indicating a beneficial effect of inhibiting viral lytic replication on the incidence of KS in AIDS patients [32] this suggests that lytic gene products may contribute to the pathogenesis of this disease. On the one hand, lytic replication can be a source of new virions and consequently newly infected cells. This is important, as KSHV does not completely immortalize spindle cells and needs to infect new cells to persist in an infected host [33]. On the other hand, lytic reactivation may lead to the production of autocrine and paracrine signalling molecules, which then promote inflammation, angiogenesis, and invasiveness. KSHV-infected endothelial spindle cells have been shown to have invasive properties [16], [20], [69], [84]–[86]. In order to better understand how KSHV lytic replication cycle contributes to the increased invasiveness of infected endothelial spindle cells we investigated cellular mechanisms underlying the lytic switch of the virus. In contrast to earlier studies that had employed siRNA screens of the human kinome to identify cellular kinases involved in KSHV reactivation and had identified Pim kinases as activators of lytic replication [44], or Tousled-like kinases as negative modulators of KSHV reactivation [87], we screened a library of small molecule kinase inhibitors (**figure 1A**) to identify positive regulators of KSHV lytic cycle. We found several compounds, known to target p38 MAPK, to inhibit KSHV reactivation after baculovirus RTA and Na-butyrate treatment (**figure S1A**), in line with previous reports on a role of p38 during *de novo* infection [88], after induction of productive reactivation [43], and during progression of KSHV through the lytic cycle, when, for instance, vGPCR activates p38 [89]. However, a close comparison of well-characterized p38 inhibitors [90]–[94], showed that these compounds varied with regard to their ability to inhibit KSHV reactivation in endothelial cells, while showing comparable efficacy in inhibiting the phosphorylation of the p38 MAPK target MK2 (**figure 1B**). This observation suggested that some of these compounds might also target other cellular kinases, which could contribute to KSHV reactivation. Off-target effects of other kinase inhibitors are well known and sometimes improve the biological activity and clinical usefulness of individual compounds [63], [95]. Since compound SB220025, which is known to have anti-inflammatory properties, proved to be the most efficient in reducing KSHV lytic reactivation (**figure 1B–C**), we profiled this substance together with VI18802, a derivative of SB220025, against 442 kinases using the KINOMEscan platform (DiscoverX). Extending previous reports on the ability of even “specific” p38 inhibitors to bind to other kinases [63], we found a range of other kinases to be inhibited by SB220025 (**figure S1B**).

By blocking, among others, the p38 cascade, SB220025 inhibits the production of IL-1β and TNF-α [91], [96], and belongs to the CSAID class of cytokine biosynthesis inhibitors [97], [98]. However, p38 is not the only regulator of inflammatory cytokine production. JNKs also regulate the expression and activation of inflammatory mediators, including TNF-α, IL-2, and MMPs [99], [100]. Interestingly, we identified several JNK isoforms and their putative upstream activators MAP4K4 (NCK interacting kinase (NIK) or haematopoietic/germinal centre kinase (HGK)), MINK (Misshapen/NIK related kinase), and TNIK (TRAF2 and NCK interacting kinase) as targets of SB220025 (**figure S1B**). KSHV is known to activate the JNK pathway during primary infection [101], and JNK is essential for KSHV infection [58], and production of inflammatory cytokines [31], [101], [102]. Considering the important role of inflammation in KS development and progression, and the dependence of KSHV on the JNK pathway, we investigated if upstream regulators of JNK signalling targeted by SB220025 (MAP4K4/NIK, TNIK, MINK) are also critical for KSHV lytic cycle. While siRNA-mediated knockdown of TNIK and MINK did not affect KSHV reactivation (data not shown), MAP4K4 silencing reduced KSHV virus production (**figure 2A**), lytic protein expression (**figure 2B**), and KSHV replication (**figure 2C**) in immortalized, as well as primary endothelial cells (**figure 7D**). Interestingly, vIL-6 expression levels were increased after MAP4K4 silencing (**figure 2B**). Although vIL-6 is a lytic gene induced by RTA [103], it is known to be also regulated independently of the lytic switch, for instance by interferon-α [66] and microRNAs, such as miR-1293 [104]. Whether MAP4K4 also regulates the latter factors needs to be further investigated, and perhaps would explain the observed increase in vIL-6 expression in the absence of MAP4K4.

As MAP4K4 was previously shown to be overexpressed in multiple tumour cell lines and cancers [49]–[51], [105], [106], and also implicated in tumour cell invasiveness [49], [106], we investigated its role in previously reported invasiveness of KSHV-infected endothelial cells. We could observe that KSHV-infected immortalized endothelial cells possess a much more invasive phenotype after the induction of the lytic cycle (**figure 3A–B**). This increased invasiveness could be reduced by MAP4K4 silencing using siRNA (**figure 3C–E**), demonstrating a role of MAP4K4 in invasive KSHV-infected endothelial cells. Similarly, silencing of MAP4K4 reduced the increased invasiveness of KSHV-infected primary umbilical vein endothelial cells (**figure 7A–C**).

The role of MAP4K4 in different cellular functions is only incompletely understood. In order to identify genes, regulated by MAP4K4 in the context of KSHV lytic reactivation, we performed a microarray-based analysis after silencing MAP4K4 and inducing the lytic cycle.

Among cellular genes known to affect migration/invasion, we found *PTGS2*, encoding COX-2, to be downregulated after MAP4K4 knockdown (**figure 4A**). Its mRNA and protein levels were highly upregulated in induced KSHV-infected cells compared to uninfected cells (**figure 4A**). Increased levels of PGE_2_ in Kaposi's sarcoma tissue compared to surrounding tissues were reported already in 1992 [107]. In keeping with this observation, KSHV infected immortalized dermal microvascular endothelial cells display a strong increase in COX-2 expression and PGE_2_ production early during *de novo* infection [20], [23], [24], when lytic replication may still take place [108]. Our finding suggests that COX-2 activation is, at least in part, mediated by MAP4K4 and is critical for KSHV lytic cycle progression, as treatment with a specific COX-2 inhibitor NS-398 (**figure 6E**) or COX-2 depletion (**figure 6B**) led to a dramatic decrease in expression of KSHV lytic proteins. COX-2 inhibitors are known to also block human cytomegalovirus replication [109], [110], as PGE_2_ enhances, for instance, CMV promoter activation [111]. COX-2 activation might also play a role in HHV-6 [112], MHV-68 [113], and HSV-1 [114] replication. Of note, MAP4K4 expression levels after COX-2 depletion and its chemical inhibition were slightly reduced (**figure 6B, 6D–E**). MAP4K4 expression is regulated by TNF-α through TNF receptor α [53], the expression levels of which in turn depend on PGE_2_ activation [115]. Hence it is conceivable that, when PGE_2_ production is downregulated by chemical inhibition of COX-2, MAP4K4 levels can also decrease as expression of TNF receptors is reduced.

COX-2 is a known mediator of angiogenesis and tumour cell invasiveness, as it leads to production of inflammatory cytokines, growth factors, angiogenic factors, and MMPs in various tumours, as well as in KSHV infected cells [20], [77], [116]–[122]. We could also show that, similarly to MAP4K4 knockdown, COX-2 silencing or chemical inhibition significantly reduces the invasiveness of KSHV-infected endothelial cells (**figure 6A, 6C**).

We also found that MAP4K4 mediates the expression of MMP-7 and MMP-13 (**figure 4A–B**), which both contribute to the invasiveness of KSHV-infected cells (**figure 5A**). Although matrix metalloproteinases are known to be modulated post-transcriptionally [123]–[125], most of them, including MMP-7 and MMP-13, can be activated also at the transcriptional level, as their promoters harbour several *cis*-elements, allowing activation by *trans*-activators, *e.g.* NF-κB and AP-1 [126], [127]. These MMPs can also be induced at the mRNA level by TNF-α, IL-1 and other cytokines [61], [128]–[131]. Given that MAP4K4 regulates inflammatory cytokine production, such as TNF-α and IL-1β [47], it may therefore also modulate MMP-7 and MMP-13 mRNA expression. Our observation that MAP4K4 regulates MMP-7 and MMP-13 expression illustrates its multifactorial role in the increased invasiveness of KSHV-infected endothelial cells.

Given the reported role of MAP4K4 as an upstream activator of JNK [64], and the role of JNK in KSHV reactivation [43], we also explored if silencing of MAP4K4 in KSHV-infected endothelial cells would alter the levels of JNK 1/2/3 phosphorylation, using phospho-specific antibodies in Western blot analysis. However, we could not detect any prominent effect of MAP4K4 silencing on the levels of JNK phosphorylation (**figure S3A**), consistent with an earlier report [47]. Searching for other cellular targets that would be phosphorylated in response to MAP4K4, we employed a commercial phosphokinase array and noted a moderate decrease of c-Jun phosphorylation following MAP4K4 silencing (**figure S3B–C**). This was confirmed in Western Blot analysis using an antibody to c-Jun phosphorylated on S63 (**figure S3D–E**). Phosphorylation of c-Jun may therefore provide another explanation of how the upstream kinase MAP4K4 exerts its effect on MMP-7 and MMP-13 expression. It might also lead to COX-2 overexpression. However, other possibilities remain to be investigated, as well as the mechanism of how MAP4K4 is activated in KSHV-infected endothelial cells.

Having shown that MAP4K4-dependent signalling pathways are involved in the increased invasiveness of KSHV-infected primary and immortalized endothelial cells, we could demonstrate that MAP4K4 is highly expressed in the pathognomonic KSHV-infected endothelial spindle cells in KS lesions (**figure 8A–D**), suggesting that it may indeed play a role *in vivo* in aspects of KSHV-induced pathogenesis. Our findings also provide an explanation for the increased expression of COX-2 in KSHV-infected endothelial cells.

## Materials and Methods

### Ethics statement

The use of the human biopsies and human umbilical cords for this study was approved by the Hannover Medical School Ethics Committee and conducted in accordance with the Declaration of Helsinki. Written informed consent was obtained from all patients.

### Cells and transfections

HEK293 and EA.hy 926 cells were maintained in Dulbecco's modified Eagle's medium (DMEM), and Vero cells in minimal essential medium (MEM) (Cytogen) supplemented with 10% foetal bovine serum (HyClone), 50 U/ml penicillin, and 50 µg/ml streptomycin (Cytogen) at 37°C in a 5% CO_2_ incubator. Human umbilical vein endothelial cells (HUVEC) were isolated from freshly obtained human umbilical cords by collagenase digestion of the interior of the umbilical vein as described previously [132] and were cultured in EGM-2MV medium (Lonza) at 37°C in a 5% CO_2_ incubator. An endothelial cell line HuAR2T, conditionally immortalized with doxycycline dependent human telomerase reverse transcriptase (hTERT) and simian virus 40 (SV40) large T antigen transgene expression [133], were maintained in EGM-2MV medium in the presence of 200 ng/ml doxycycline. Transfection with small interfering RNA (siRNA) was performed using the Neon transfection system according to the manufacturer's instructions (Invitrogen). All siRNAs were microporated at the concentration of 100 pmol into 10^5^ cells. The following siRNAs (siGENOME SMARTpool) were obtained from Dharmacon, Thermo Scientific: Control (Non-targeting siRNA Pool #2, D-001206-14-20), MAP4K4 (M-003971-02-0005), MMP-7 (M-003782-01-0005), MMP-13 (M-005955-01-0005).

Sf9 cells were maintained in Grace's medium (Gibco) supplemented with 10% foetal bovine serum, 100 U/ml penicillin, and 50 µg/ml streptomycin (Cytogen) at 28°C. The generation of the recombinant baculovirus expressing KSHV ORF50/RTA was described previously [60].

### Production of recombinant KSHV stocks

To produce virus stocks, Vero cells containing recombinant KSHV (rKSHV.219) [60] were plated at 30–40% confluency in T175 flasks and induced twenty-four hours later with 1 mM Na-butyrate (Sigma-Aldrich) and 10% baculovirus coding for KSHV ORF50/RTA. The supernatant was harvested 72 hours later and 0.45 µm filtered to remove cell debris. The cleared supernatant was collected in centrifuge bottles (230 ml/bottle) and centrifuged at 15000×g at 4°C for 6 hours using a Type19 rotor (Beckman Coulter). The supernatant was then discarded and the pellet resuspended in 250 µl EBM2 basal medium (Lonza) overnight at 4°C. The resuspended virus was kept at 4°C for not longer than three weeks.

For detection and quantification of KSHV titres, 2.3×10^4^ HEK293 cells were plated in a 96-well plate and infected with serially diluted KSHV stocks. GFP-positive cells were counted two days after infection. To determine KSHV titres from EA.hy rKSHV.219 or HuAR2T rKSHV.219 cells after induction of the lytic cycle, the supernatants were cleared from the debris by 0.45 µm filtration and applied to HEK293 cells without dilution.

### Western blot analysis

Protein lysates of cells were prepared in 1× SDS sample buffer (62.5 mM Tris-HCl pH 6.8, 2% w/v SDS, 10% glycerol, 50 mM DTT, 0.01% w/v bromophenol blue) supplemented with cOmplete Ultra protease inhibitor cocktail and PhosSTOP phosphatase inhibitor cocktail (Roche). Proteins were resolved by SDS-PAGE, transferred onto nitrocellulose membranes (GE Healthcare), and detected using the following primary antibodies: rabbit polyclonal MAP4K4 (HGK) antibody (#3485, Cell Signaling Technology), rabbit polyclonal KSHV ORF50/RTA [37], mouse monoclonal KSHV ORF45 antibody (sc-53883, Santa Cruz), mouse monoclonal HHV-8 KbZIP antibody F33P1 (sc-69797, Santa Cruz), rabbit polyclonal KSHV vIL-6 antibody (13-214-050, Advanced Biotechnologies), mouse monoclonal KSHV ORFK8.1A/B antibody (13-212-100, Advanced Biotechnologies), mouse monoclonal β-actin antibody (A5441, Sigma-Aldrich), rabbit monoclonal GAPDH antibody (#2118, Cell Signaling Technology), rabbit polyclonal COX-2 antibody (#4842, Cell Signaling Technology), rat monoclonal KSHV ORF73 (LNA-1) antibody (13-210-100, Advanced Biotechnologies), mouse monoclonal phospho-JNK 1/2/3 antibody 9H8 (sc-81502, Santa Cruz), rabbit polyclonal JNK 1/3 antibody C17 (sc-474, Santa Cruz), mouse monoclonal phospho-p44/42 antibody (#9106, Cell Signaling Technology), mouse monoclonal p44/p42 antibody 3A7 (#9107, Cell Signaling Technology), rabbit polyclonal phospho-p38 antibody (#9211, Cell Signaling Technology), rabbit polyclonal p38 antibody (#9212, Cell Signaling Technology), rabbit monoclonal phospho-MK2 antibody 27B7 (#3007, Cell Signaling Technology), rabbit polyclonal MK2 antibody (#3042, Cell Signaling Technology). All stainings were performed at 4°C overnight with subsequent washing in TBS-T or PBS-T and incubation with a corresponding secondary HRP-labelled antibody (DaKo) at RT for one hour. Following further washing steps, the proteins were detected with SuperSignal West Femto Chemiluminescent Substrate (Pierce, Thermo Scientific).

### Microarray-based mRNA expression analysis

The “Whole Human Genome Oligo Microarray V2” (G4845A, ID 026652, Agilent Technologies) used in this study contains 44495 oligonucleotide probes covering roughly 27390 human transcripts. Synthesis of Cy3-labeled cRNA was performed with the “Quick Amp Labelling kit, one colour” (#5190-0442, Agilent Technologies) according to the manufacturer's recommendations. cRNA fragmentation, hybridization, and washing steps were carried out exactly as recommended in the “One-Color Microarray-Based Gene Expression Analysis Protocol V5.7” (Agilent). Slides were scanned on the Agilent Micro Array Scanner G2565CA (pixel resolution 5 µm, bit depth 20). Data extraction and processing of raw fluorescence intensity values were performed with the “Feature Extraction Software V10.7.3.1” by using the recommended default extraction protocol file: GE1_107_Sep09.xml.

Processed intensity values of the green channel (“gProcessedSignal” or “gPS”) were globally normalized by a linear scaling approach: All gPS values of one sample were multiplied by an array-specific scaling factor. This scaling factor was calculated by dividing a “reference 75th Percentile value” (set as 1500 for the whole series) by the 75th Percentile value of the particular Microarray (“Array i” in the formula shown below). Accordingly, normalized gPS values for all samples (microarray data sets) were calculated by the following formula: normalized gPSArray i = gPSArray i X (1500/75th PercentileArray i). A lower intensity threshold was defined as 1% of the reference 75th Percentile value ( = 15). All normalized gPS values below this intensity threshold were substituted by the surrogate value of 15.

Data were filtered according to the following criteria: 1) More than 1.5 fold downregulation in lytically induced HuAR2T rKSHV.219 cells after MAP4K4 knockdown compared to control siRNA treated induced cells (each of three experiments). 2) Arithmetic mean intensity of nPS values calculated from both channels that define ratio values >25 (each of three experiments). 3) QC flag entries “gIsNonUnifOL” (determined by the Feature Extraction Software) must have been “0” (indicating reliable performance) (each of six relevant channels of the three experiments). 4) In cases, in which more than one probe directed against the same transcript is present on the microarray, only those transcripts passed the criteria, for which the majority of probes indicate the respective regulation. 5) The respective transcript has to be classified as being functionally characterized and reasonably annotated (for details visit: www.mh-hannover.de/Transcriptomics.html and consult our manual: “Crude probe characterization_RCUT_date.pdf”). Just one representative probe is selected for visualization in **figure 4A** if many probes directed against the same transcript match the applied criteria.

### q(RT)-PCR analysis

Total RNA was extracted from the cells with an RNeasy kit (QIAgen) according to the manufacturer's recommendations, followed by DNase treatment and inactivation (Ambion). cDNA was synthesized using BioScript RNase H Low reverse transcriptase (BIO-27036, Bioline) or Expand reverse transcriptase (Roche) in 20 µl reactions. 1 µl of generated cDNA samples (50 ng total RNA equivalents) were used per reaction for real-time PCR with the ABI7500 system (Applied Biosystems). Specific amplification was assured by utilizing TaqMan probes and gene specific primers. Amplification was performed in 10 µl reactions with TaqMan Fast Advanced Master Mix under recommended conditions (Applied Biosystems; #4444557). The following TaqMan gene expression assays (Applied Biosystems: #4331182) were used: Hs00153133_m1 (PTGS2/COX-2), Hs99999908_m1 (GUSB), Hs01042796_m1 (MMP-7), Hs00233992_m1 (MMP-13), Hs02758991_g1 (GAPDH); primer-probe sets for RTA [134], KbZIP [70], K8.1 [135]. The average Ct for each individual amplification reaction was calculated from duplicate measurements by means of the instrument's software in “auto Ct” mode (7500 System Software v.1.3.0). Average Ct values obtained for the analysed transcripts of PTGS2/COX-2, MMP-7 or MMP-13 were normalized by subtraction from the Ct values obtained for GUSB or GAPDH (housekeeping reference). Relative mRNA expression changes were calculated according to the ΔΔCt method.

For quantification of KSHV genome copies, DNA was extracted using the QIAamp DNA Blood Mini Kit (QIAgen) according to the manufacturer's instructions. KSHV genome copy numbers were determined in a TaqMan based qPCR directed against KSHV ORF K6 with normalization to the cellular C-reactive protein (CRP) as described previously [136]. Briefly, qPCR for KSHV was carried out in a total volume of 50 µl containing a ready-to-use master mix (QuantiTect multiplex PCR kit; Qiagen), 0.5 µM concentrations of each primer, 10 µl of DNA from the sample of interest, and 0.4 µM FAM-labeled KSHV K6 probe. Amplification was performed in the Applied Biosystems 7500 thermal cycler and visualized with ABI 7500 software. qPCR of CRP was carried out in a total volume of 20 µl containing a ready-to-use master mix (LightCycler FastStart DNA Master HybProbe; Roche), 0.3 mM MgCl2, 0.5 µM concentrations of each primer, 0.2 µM FAM-labeled CRP probe, and 5 µl of DNA from the sample of interest. Amplification was performed in the LightCycler 2.0 Instrument and analyzed with the LightCycler software. The primers (Sigma) and probes (Eurogentec) used for the quantification of KSHV and CRP had the following sequences: KSHV K6 forward (CGCCTA ATAGCTGCTGCTACGG), HHV8 K6 reverse (TGCATCAGCTGCCTAACCCAG), CRP forward (CTTGACCAGCCTCTCTCATGC), CRP reverse (TGCAGTCTTAGACCCCACCC), K6 probe [5′-(6 FAM)-CAGCCACCGCCCGTCCAAATTC-TAMRA], and CRP probe [5′-(6 FAM)-TTTGGCCAGACAGGTAAGGGCCACC-TAMRA].

### *In vitro* invasion assay

Cell invasiveness was measured using Matrigel coated invasion inserts (Growth Factor Reduced Matrigel Invasion Chamber, 8.0 µm; 354483, BD Biosiences). HuAR2T rKSHV.219 cells were microporated with siRNAs twenty hours prior to the induction of the lytic cycle. Twenty-four hours after the induction, the cells were starved in EBM2 supplemented with 2% FCS for twelve hours. Next day, 5×10^4^ cells were plated in the inner chambers in 500 µl of EBM2 basal medium with 2% FCS and 750 µl EBM2 was added to the outer chamber and incubated for twenty-four hours. Before the assay, Matrigel inserts were rehydrated with 500 µl EBM2 for two hours. Cells that were able to degrade the Matrigel layer, migrated to the lower surface of the filter, and were fixed with 4% paraformaldehyde, permeabilised with 0.2% Triton X-100, and nuclei were stained with DAPI (Sigma-Aldrich) and counted under a fluorescent microscope. Four different Matrigel chambers were used for each sample. Four random fields were counted for each chamber, and the average cell number per field in a chamber was calculated using CellProfiler2.0. To quantify the number of cells in the immunofluorescence images we used the CellProfiler software [137]. All pixel intensities were rescaled to 0–1. Using the Otsu Global thresholding method [138] in the DAPI channel, the nuclear area was defined. Clumped nuclei were distinguished based on the intensity. The threshold correction factor was set to 1.3. Cell invasiveness of freshly isolated HUVECs (<p. 2) was measured after infection with rKSHV.219 at MOI 30 for four days. Three days after infection the cells were microporated with siRNAs and starved in EBM2 medium with 2% FCS. After twenty-four hours the cells were seeded onto the Matrigel, and their invasiveness was quantified as described above.

### Immunohistochemistry

3 µm thin tissue sections were cut from formalin-fixed KS samples and stained with haematoxylin-eosin (HE). KS tumour cells and non-neoplastic endothelial cells were marked immunohistochemically with anti-CD34 antibody (Menarini Corp.) using a 1∶50 dilution. The KSHV latent nuclear antigen (LANA) was marked immunohistochemically with NCL-HHV8-LNA antibody, clone 13B10, purchased from Novocastra, using a 1∶50 dilution. MAP4K4 was stained immunohistochemically with MAP4K4 monoclonal antibody M07, clone 4A5, produced by Abnova Corp. and purchased from Biozol Diagnostica GmbH, applied at a 1∶300 dilution. When performing MAP4K4/LANA double-staining, 1∶20 (LANA) and 1∶100 (MAP4K4) dilutions were applied using the BenchMark Ultra staining machine.

### Small molecule inhibitors and KINOME*scan*

The library of kinase inhibitors was received from Vichem Chemie Research Ltd. (Budapest, Hungary) as lyophilized powders, and stored at room temperature. DMSO (cell culture grade, Applichem) was used to dissolve the inhibitors at a stock concentration of 10 mM. After reconstitution, the inhibitors were stored at room temperature protected from light. VI18802 is a phenoxypyrimidine [93] targeting p38α. As a part of the Vichem Core Validation Library it was handled as described above. SB203580 (#13067, Cayman Chemical), SB202190 (#EI-294-0001, Biomol), SB220025 (#559396, Calbiochem), SKF86002 (#2008, Tocris Bioscience), and VX-745 (#3915, Tocris Bioscience) were reconstituted and stored in working aliquots at −20°C protected from light. To target COX-2, NS-398 (#349254, Calbiochem) was prepared according to the manufacturer's recommendations and applied to the cells six hours before the induction of the lytic cycle. Application of all compounds to the cells was controlled by DMSO treatment.

For evaluation of KSHV reactivation inhibition, 5×10^3^ Vero rKSHV.219 cells per well were plated in 96-well plates twenty-four hours before the treatment with kinase inhibitors. The inhibitors were applied one hour before the induction of the lytic cycle. Forty-eight hours later the supernatants were transferred to HEK293 cells, which were then centrifuged 30 min at 30°C and 500×g and incubated at 37°C for six hours. Medium was exchanged, and the cells were incubated at 37°C for forty-eight hours. The number of infectious particles was evaluated by the mean fluorescence intensity of GFP-positive HEK293 cells. Alternatively, Vero rKSHV.219 or EA.hy rKSHV.219 cells were treated with kinase inhibitors, and subsequently with induction mix to assess the expression levels of KSHV lytic proteins RTA and K8.1.

To assay the viability of cells after treatment with kinase inhibitors, each well of a 96-well plate received 20 µl glutaraldehyde (25%) and was incubated at RT for at least 20 min. After washing with water, the plates were stained with 0.4% crystal violet solution in methanol for 30 min. Absorbance at 590 nm was measured spectrophotometrically with a reference to 405 nm reading.

The KINOME*scan* of SB220025 and VI18802 was carried out by DiscoverX as described (www.discoverx.com).

### Analysis of Ser/Thr kinase phosphorylation profiles

For evaluation of Ser/Thr kinase phosphorylation in endothelial cells, a human phospho-kinase antibody array (ARY003B, R&D Systems) was used according to the manufacturer's recommendations. Briefly, HuAR2T rKSHV.219 cells were transfected with control siRNA or an siRNA pool targeting MAP4K4 twenty-four hours before the induction of the lytic cycle. Cells were lysed twenty-four after lytic cycle induction and diluted lysates were applied to, and incubated overnight with, the nitrocellulose membranes with spotted capture antibodies. The array was washed to remove unbound proteins, followed by incubation with a cocktail of biotinylated detection antibodies, and streptavidin-HRP. Chemiluminescent detection reagents (SuperSignal West Femto Chemiluminescent Substrate, 34096, Thermo Scientific) were applied as recommended. The signal produced at each capture spot corresponded to the amount of phosphorylated protein bound.

### Statistical analysis

Statistical analysis was performed using GraphPad prism software. For the comparison of more than two groups a one-way-ANOVA with Tukey's post-test was applied after using D'Agostino-Pearson's normality test where applicable. P-values <0.05 were considered as significant (*), <0.01 (**), <0.001 (***), and <0.0001 (****). P-values >0.05 were considered non-significant (ns). Error bars were calculated from means ±SD. The qPCR data are shown as means ±SEM, where one replicate is shown as a representative.

### Accession numbers

Akt (P31749), b-Raf (P15056), CD34 (P28906), CDC2L1 (A4VCI5), c-Jun (P05412), COX-1 (P23219), COX-2 (P35354), CSNK1A1L (Q8N752), CSNK1D (P48730), CSNK1E (P49674), ERK1 (P27361), IFN-α (P01562), IL-1β (P01584), IL-2 (P60568), IL-6 (P05231), JNK1 (P45983), JNK3 (P53779), KSHV K15 (Q9QR69), KSHV K8.1 (D2XQF0), KSHV KbZIP (E5LBX3), KSHV LANA (J9QT20), KSHV ORF45 (F5HDE4), KSHV RTA (F5HCV3), KSHV v-Cyclin (Q77Q36), KSHV v-FLIP (Q76RF1), KSHV vGPCR (Q98146), KSHV vIL-6 (Q98823), MAP4K4 (O95819), MEK1 (Q02750), MINK (Q8N4C8), MK2 (P49137), MMP-1 (P03956), MMP-13 (P45452), MMP-19 (Q99542), MMP-2 (P08253), MMP-3 (P08254), MMP-7 (P09237), MMP-9 (P14780), NF-κB (Q04206), Notch (P46531), p38α (Q16539), PI3K (P42336), Pim-1 (P11309), Pim-3 (Q86V86), PKA (P17612), PKCδ (Q05655), RBP-Jκ (Q06330), STK36 (Q9NRP7), TLR7/8 (D1CS68), TNF-α (P01375), TNFR α (P19438), TNIK (Q9UKE5).

## Supporting Information

Figure S1 **Effect of p38 inhibitors on KSHV production and lytic protein expression.** (A) Summary of 18 compounds able to efficiently block KSHV production and late lytic protein K8.1 expression in VK.219, BCBL1 and EA.hy rKSHV.219 cells. (B) Identification of targets for SB220025 and VI18802 using the KINOMEscan profiling platform. Target list of SB220025 and VI18802 identified in this study, as compared to SB202190, SB203580, and VX745 [63]. Unique targets for SB220025 and VI18802 are presented in grey fields.
(PDF)

Figure S2 **Efficiency of MAP4K4 knockdown and its effect on baculovirus RTA delivery.** (A) Western blot analysis of MAP4K4 and K8.1 expression levels in HuAR2T rKSHV.219 cells, transfected with control siRNA, an siRNA pool or individual siRNAs targeting MAP4K4, and induced to reactivate KSHV for forty-eight hours. (B) Schematic of individual siRNAs targeting MAP4K4. (C) qPCR analysis of *RTA* expression from baculovirus transduced into uninfected HuAR2T cells. The graph shows means ±SD of three independent experiments. The p values were determined using a One-way ANOVA with Tukey's multiple comparison post-test. p>0.05 (ns). (D) Western blot analysis of MAP4K4 expression after siRNA silencing in uninfected HuAR2T transduced with baculovirus coding for RTA. The blot is one representative of two independent experiments with similar results. (E) Efficiency of MAP4K4 knockdown in cells used for microarray based gene expression analysis. HuAR2T rKSHV.219 cells were transfected with control siRNA or an siRNA pool targeting MAP4K4 twenty-four hours before the induction of the lytic cycle. Twenty-four hours after the lytic cycle induction cells were harvested and lysed for subsequent analysis of protein expression. Presented are the Western blots for MAP4K4 and KSHV lytic protein expression.
(PDF)

Figure S3 **Effect of MAP4K4 knockdown on putative downstream phosphorylation targets.** HuAR2T rKSHV.219 cells were transfected with control siRNA or an siRNA pool targeting MAP4K4, twenty-four hours before the induction of the lytic cycle. Twenty-four to forty-eight hours after the lytic cycle induction cells were harvested and lysed for subsequent analysis of protein phosphorylation and expression. (A) Western blot analysis of phosphorylated species of the indicated proteins performed forty-eight hours after lytic cycle induction. (B) Analysis of phosphorylation of Ser/Thr kinases with a human phospho-kinase antibody array twenty-four hours after lytic cycle induction. Positive and negative controls are encircled, and a potential downstream target of MAP4K4 – c-Jun – is marked with an arrow. (C) Quantification of results obtained with phospho-kinase array. The signals from arrays as shown in panel B were quantified in ImageJ and are presented as a mean ±SD of the corresponding capture spots. (D) Verification of MAP4K4 knockdown efficiency in the cell lysates used for the human phospho-kinase analysis. (E) Validation of the identified potential downstream target of MAP4K4 by Western blot analysis of Ser63 c-Jun phosphorylation in HuAR2T rKSHV.219 twenty-four hours after lytic cycle induction.
(PDF)
