# Supplementary material for: The Inflammatory Kinase MAP4K4 Promotes Reactivation of Kaposi's Sarcoma Herpesvirus and Enhances the Invasiveness of Infected Endothelial Cells
Source: PLoS Pathog. 2013 Nov 7;9(11):e1003737. doi: 10.1371/journal.ppat.1003737 (PMC3820715; doi:10.1371/journal.ppat.1003737)
Supplement: Figure S1 — Effect of p38 inhibitors on KSHV production and lytic protein expression. (A) Summary of 18 compounds able to efficiently block KSHV production and late lytic protein K8.1 expression in VK.219, BCBL1 and EA.hy rKSHV.219 cells. (B) Identification of targets for SB220025 and VI18802 using the KINOMEscan profiling platform. Target list of SB220025 and VI18802 identified in this study, as compared to SB202190, SB203580, and VX745 [63]. Unique targets for SB220025 and VI18802 are presented in grey fields. (PDF) [file ppat.1003737.s001.pdf]

A

Vichem ID	Commercial ID (if available)	Known target	Virus production inhibition, IC50 VK.219	K8.1 expression inhibition VK.219	BCBL1	EA.hy rKSHV.219
VI7008	SB203580 der.	p38	2-5 µM	75% 5 µM	25% 5 µM	25% 2 µM
VI8031	PD166326 der.	PDGFR, Src	< 5 µM	75% 10 µM	25% 10 µM	50% 5 µM
VI9075	1,6-Naphtyridine-2-carboxamide der.	Cytomegalovirus inhibitor	1-2 µM	25% 2 µM	25% 2 µM	25% 2 µM
VI9689	SU5416 der.	EGFR, FLK	< 5 µM	25% 10 µM	75% 10 µM	50% 5 µM
VI9925	BIRB796 der.	p38	5-10 µM	25% 5 µM	75% 2 µM	25% 5 µM
VI11877	9-Trifluoro-methylpaullone	CDK	1 µM	25% 2 µM	25% 2 µM	75% 2 µM
VI13786	Brequinar der.	DHODH	2 µM	50% 2 µM	50% 10 µM	50% 2 µM
VI15277	ABT702 der.	AdK	1-2 µM	25% 10 µM	75% 10 µM	25% 10 µM
VI16118	Quinilinyl-pyrazole der.	p38	2-5 µM	50% 5 µM	50% 5 µM	25% 5 µM
VI16339	VX745	p38	0.5-1 µM	25% 2.5 µM	75% 10 µM	50% 2 µM
VI17423	SKI606	Src	5-10 µM	50% 10 µM	50% 10 µM	75% 10 µM
VI18280	SB220025 der.	p38	0.5-1 µM	25% 2 µM	25% 2 µM	25% 2 µM
VI18281	SB220025 der.	p38	0.5-1 µM	25% 2 µM	25% 2 µM	50% 2 µM
VI18554	SB220025 der.	p38	< 0.5 µM	50% 2 µM	75% 10µM	75% 2 µM
VI18661	SB220025 der.	p38	0.5-1 µM	25% 2 µM	50% 2 µM	75% 2 µM
VI18662	SB220025 der.	p38	0.5-1 µM	50% 2 µM	50% 2 µM	75% 2 µM
VI18802	SB220025 der.	p38	1 µM	50% 2 µM	75% 1 µM	25% 2 µM
VI18803	SB220025 der.	p38	0.5-1 µM	25% 2 µM	25% 2 µM	25% 2 µM

B

<table>
<tr><th>VI18802</th><th>SB220025</th><th>SB203580</th><th>SB202190</th><th>VX745</th></tr>
<tr><td colspan="5">p38alpha
p38beta</td></tr>
<tr><td colspan="4">CIT
CSNK1D
CSNK1E
DMPK2
JNK2
JNK3</td><td rowspan="6"></td></tr>
<tr><td>EGFR</td><td></td><td>EGFR</td><td>EGFR</td></tr>
<tr><td rowspan="3"></td><td colspan="3">GAK</td></tr>
<tr><td colspan="3">NLK</td></tr>
<tr><td colspan="3">RIPK2</td></tr>
<tr><td colspan="2">CSNK1A1L
MINK
MRCKA
MRCKB
YANK1
YANK2</td><td colspan="2" rowspan="3"></td></tr>
<tr><td>ERBB2
ERK2</td><td></td></tr>
<tr><td></td><td>CDC2L1
CDC2L2
CSNK1G2
CSNK1G3
DDR1
JNK1
MAP4K4
p38delta
PKACalpha
PRKD2
PRKD3
RSK1
RSK4
STK36
TNIK</td><td></td></tr>
</table>
