# Supplementary figures and images for: The Inflammatory Kinase MAP4K4 Promotes Reactivation of Kaposi's Sarcoma Herpesvirus and Enhances the Invasiveness of Infected Endothelial Cells
Source: PLoS Pathog. 2013 Nov 7;9(11):e1003737. doi: 10.1371/journal.ppat.1003737 (PMC3820715; doi:10.1371/journal.ppat.1003737)

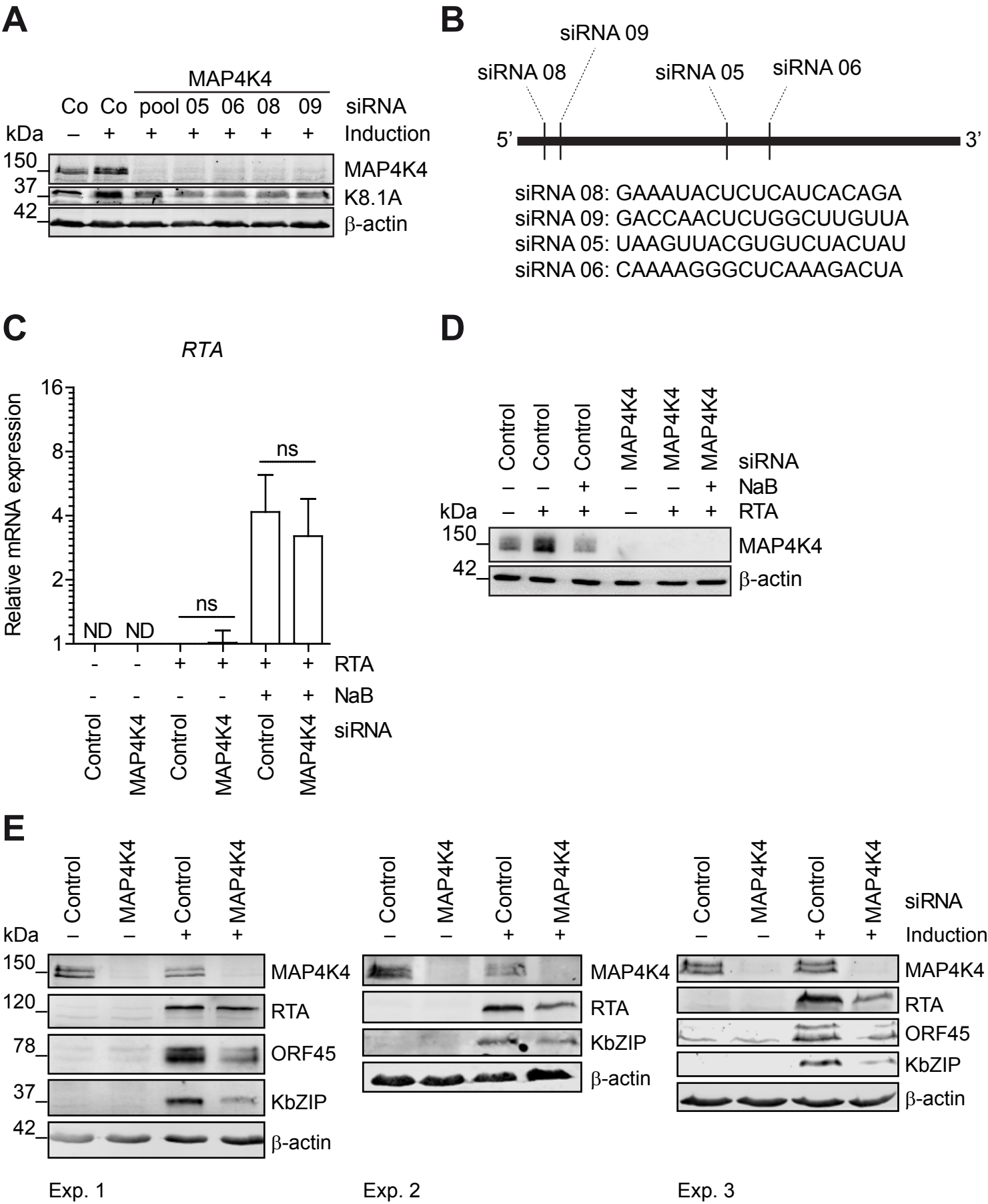

Supplement: Figure S2 — Efficiency of MAP4K4 knockdown and its effect on baculovirus RTA delivery. (A) Western blot analysis of MAP4K4 and K8.1 expression levels in HuAR2T rKSHV.219 cells, transfected with control siRNA, an siRNA pool or individual siRNAs targeting MAP4K4, and induced to reactivate KSHV for forty-eight hours. (B) Schematic of individual siRNAs targeting MAP4K4. (C) qPCR analysis of RTA expression from baculovirus transduced into uninfected HuAR2T cells. The graph shows means ±SD of three independent experiments. The p values were determined using a One-way ANOVA with Tukey's multiple comparison post-test. p>0.05 (ns). (D) Western blot analysis of MAP4K4 expression after siRNA silencing in uninfected HuAR2T transduced with baculovirus coding for RTA. The blot is one representative of two independent experiments with similar results. (E) Efficiency of MAP4K4 knockdown in cells used for microarray based gene expression analysis. HuAR2T rKSHV.219 cells were transfected with control siRNA or an siRNA pool targeting MAP4K4 twenty-four hours before the induction of the lytic cycle. Twenty-four hours after the lytic cycle induction cells were harvested and lysed for subsequent analysis of protein expression. Presented are the Western blots for MAP4K4 and KSHV lytic protein expression. (PDF) [file ppat.1003737.s002.pdf]

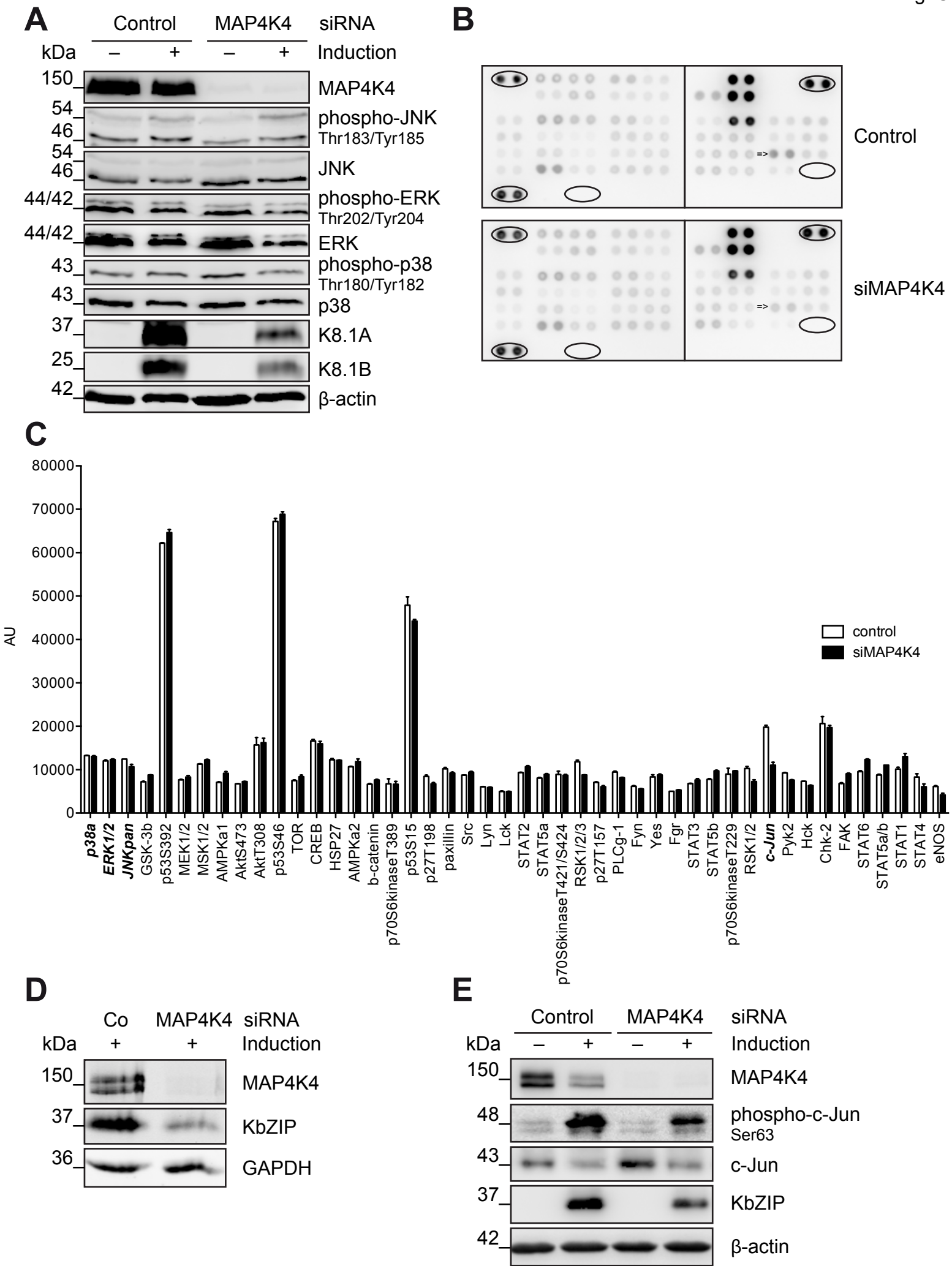

Supplement: Figure S3 — Effect of MAP4K4 knockdown on putative downstream phosphorylation targets. HuAR2T rKSHV.219 cells were transfected with control siRNA or an siRNA pool targeting MAP4K4, twenty-four hours before the induction of the lytic cycle. Twenty-four to forty-eight hours after the lytic cycle induction cells were harvested and lysed for subsequent analysis of protein phosphorylation and expression. (A) Western blot analysis of phosphorylated species of the indicated proteins performed forty-eight hours after lytic cycle induction. (B) Analysis of phosphorylation of Ser/Thr kinases with a human phospho-kinase antibody array twenty-four hours after lytic cycle induction. Positive and negative controls are encircled, and a potential downstream target of MAP4K4 – c-Jun – is marked with an arrow. (C) Quantification of results obtained with phospho-kinase array. The signals from arrays as shown in panel B were quantified in ImageJ and are presented as a mean ±SD of the corresponding capture spots. (D) Verification of MAP4K4 knockdown efficiency in the cell lysates used for the human phospho-kinase analysis. (E) Validation of the identified potential downstream target of MAP4K4 by Western blot analysis of Ser63 c-Jun phosphorylation in HuAR2T rKSHV.219 twenty-four hours after lytic cycle induction. (PDF) [file ppat.1003737.s003.pdf]
